# Multiethnic genome-wide association study identifies ethnic-specific associations with body mass index in Hispanics and African Americans

**DOI:** 10.1186/s12863-016-0387-0

**Published:** 2016-06-13

**Authors:** Yasmmyn D. Salinas, Leyao Wang, Andrew T. DeWan

**Affiliations:** Department of Chronic Disease Epidemiology, Yale School of Public Health, 60 College Street, New Haven, CT 06520 USA

**Keywords:** Obesity, Body mass index, Ethnic differences, Genetic epidemiology

## Abstract

**Background:**

Genome-wide association studies of obesity have typically assumed fixed genetic effects across ethnicities, rarely attempting to thoroughly compare and contrast findings across various ethnic groups. Therefore, our study aimed to identify novel genetic associations with body mass index (BMI), a common measure of obesity, and explore their cross-ethnic generalizability in a multiethnic population. To that end, we conducted ​ethnic-specific genome-wide association analyses among 1235 Hispanic, 706 Asian, 1549 African American, and 2395 European American subjects from the Multi-ethnic Study of Atherosclerosis (MESA). We compared findings ​across ethnicities and investigated single-nucleotide polymorphisms (SNPs) with suggestive BMI-association p-values among 3379 Hispanic and 6871 African American subjects from the Women’s Health Initiative (WHI).

**Results:**

We identified a genome-wide significant association in MESA Hispanics—rs12253976 in *KLF6* (beta = 5.792 kg/m^2^ per-allele, 95 % confidence interval (CI): 3.885, 7.698; *p* = 3.43 × 10^−9^)—and suggestive SNPs with *p* < 5 × 10^−6^ in MESA Hispanics, European Americans and African Americans that display ethnic-specific effects on BMI. Of these suggestive SNPs, Hispanic SNP rs12255372 and African American SNP rs6435678 had the most evidence of replication in WHI. rs12255372 (in *TCF7L2*) was associated with lower BMI in both MESA (beta = −1.111 kg/m^2^, 95 % CI: −1.578, −0.645; *p* = 3.33 × 10^−6^) and WHI Hispanics (beta = −0.304 kg/m^2^, 95 % CI: −0.613, 0.006; *p* = 0.054). This *TCF7L2* intronic region contains several SNPs (rs7901695, rs4506565, rs4132670, and rs12243326) with low p-values (*p* < 10^−3^) in MESA and betas of similar magnitude and direction in MESA and WHI, but only rs12243326 is in strong linkage disequilibrium with rs12255372 in our Hispanic populations, suggesting independent signals in this region. rs6435678 (in *ERBB4*) was associated with greater BMI in both MESA (beta = 1.104 kg/m^2^, 95 % CI: 0.643, 1.564; *p* = 2.85 × 10^−6^) and WHI African Americans (beta = 0.219 kg/m^2^, 95 % CI: −0.021, 0.460; *p* = 0.074).

**Conclusions:**

Two BMI-association signals are present in the *TCF7L2* intronic region of Hispanics, one of which is tagged by rs12255372. *ERBB4* rs6435678 is a novel BMI-association signal in African Americans*.* Overall, our data suggest that ethnic-specific associations are involved in the genetic determination of BMI. Ethnic-specificity has potential implications for the development of gene-based therapies for obesity.

**Electronic supplementary material:**

The online version of this article (doi:10.1186/s12863-016-0387-0) contains supplementary material, which is available to authorized users.

## Background

Obesity is one of the most pressing health problems in the United States (U.S.). It affects nearly 35 % of adults and 17 % of children [1], predisposing them to many chronic conditions including type 2 diabetes (T2D), cardiovascular disease (CVD), and several cancers [2]. The cost of treating obesity-related conditions places great financial burden on the healthcare system [3]. Consequently, understanding the etiology of obesity and developing interventions to prevent its comorbidities are critical public health concerns.

The etiology of obesity is multifactorial [4], but family studies suggest that 40-70 % of the variation in body mass index (BMI), a common measure of obesity, is explained by genetic factors [5–7]. Genome-wide association studies (GWAS), which have identified over 100 loci associated with BMI and other obesity-related traits, have greatly expanded our understanding of the genetic basis of obesity [8]. However, further investigation is warranted for several reasons. First, known BMI loci account for only a fraction of the estimated variation in BMI [9]. Second, previous GWAS have primarily relied on data from subjects of European ancestry [10]. Hispanics and African Americans are underrepresented in GWAS, and it is precisely these populations that are overburdened by obesity in the U.S. [11]. Third, obesity GWAS have either analyzed a single ethnic group in isolation or pooled multiethnic data in cross-ethnic meta-analyses, assuming that genetic effects are *fixed* across ethnic groups, and have rarely attempted to thoroughly compare and contrast findings across ethnic groups.

As noted in [12], to more fully gauge the clinical and public health implications of genetic associations with BMI, studies should not only focus on the replication of genetic loci identified in European populations; they should also evaluate the cross-ethnic generalizability of genetic associations in multiethnic populations. Making unbiased cross-ethnic comparisons of genetic effects is facilitated by the availability of data from multiple ethnic groups sampled in the same fashion from the same underlying source population. Conducting ethnic-specific GWAS within such multiethnic populations could also reveal loci not readily detectable in Europeans due to cross-ethnic differences in allele frequencies and haplotype structures [13].

For these reasons, we used an ethnic-specific GWAS approach to examine genetic associations with BMI in the Multi-ethnic Study of Atherosclerosis (MESA), which includes subjects of four ethnicities: Hispanic, Asian, African American, and European American. We identified the top BMI-associated single-nucleotide polymorphisms (SNPs) in each ethnicity and evaluated whether those SNPs were associated with BMI to a similar extent in the other ethnicities. We then sought to replicate the top SNPs in Hispanics and African Americans in an independent cohort consisting of multiethnic subjects from the Women’s Health Initiative (WHI).

## Results

### Discovery sample characteristics and model covariates

Descriptive statistics for MESA are shown in Table 1. Unadjusted associations between participant characteristics and BMI are summarized in Additional file 1: Table S1. After model building, ethnic-specific covariates were: age, sex, smoking, diabetes, and arthritis in Hispanics; age, sex, education, diabetes, and arthritis in Asians and African Americans; and age, sex, income, education, smoking, physical activity, diabetes and arthritis in European Americans.

**Table 1:** Description of the MESA study population by ethnicity ^a^

Characteristic^b^	Hispanics (*n* = 1235)^c,d^	Asians (*n* = 706)^c,d^	African Americans (*n* = 1549)^c,d^	European Americans (*n* = 2395)^c,d^
Age (years)	61.26 ± 10.27	62.50 ± 10.36	62.35 ± 10.10	62.57 ± 10.19
Sex				
Female	624 (50.5)	357 (50.6)	827 (53.4)	1246 (52.0)
Male	611 (49.5)	349 (49.4)	722 (46.6)	1149 (48.0)
Income				
Low	894 (74.0)	468 (66.7)	733 (51.2)	756 (31.6)
High	314 (26.0)	234 (33.3)	700 (48.9)	1639 (68.4)
Education				
<12 years	577 (46.7)	172 (24.4)	188 (12.1)	115 (4.8)
12-15 years	532 (43.1)	257 (36.4)	839 (54.2)	1074 (44.8)
≥16 years	126 (10.2)	277 (39.2)	522 (33.7)	1206 (50.4)
Smoking				
Ever	563 (45.6)	176 (24.9)	852 (55.0)	1331 (55.6)
Never	672 (54.4)	530 (75.1)	697 (45.0)	1064 (44.4)
Physical Activity (met-min/wk)	5939.53 ± 6002.02	3764.16 ± 3915.99	6542.21 ± 6937.06	5699.50 ± 5383.03
Diabetes				
Yes	207 (16.8)	77 (10.9)	242 (15.6)	124 (5.2)
No	1028 (83.2)	629 (89.1)	1307 (84.4)	2271 (94.8)
Arthritis				
Yes	432 (35.0)	181 (25.6)	675 (43.6)	849 (35.5)
No	803 (65.0)	525 (74.4)	874 (56.4)	1546 (64.6)
Body Mass Index (kg/m^2^)	29.35 ± 5.18	24.02 ± 3.27	30.11 ± 5.86	27.75 ± 5.07

*Abbreviation*: *met-min/wk* metabolic minutes per week

^a^ Table values are mean ± SD for continuous variables and n (column %) for categorical variables

^b^ Characteristics included are covariates in at least one ethnic-specific linear regression model

^c^ Sample size represents number of individuals that passed quality control and have complete data for all ethnic-specific linear regression model covariates

^d^ Percentages may not sum to 100 % due to rounding and n’s may not sum to sample size due to missing values for variables not included as covariates in ethnic-specific regression models

### Population stratification

Additional file 2: Figures S1-S2 shows quantile-quantile plots of observed vs. expected p-values in the discovery and replication datasets, before and after adjustment for population stratification. Before adjustment, there was evidence of genomic inflation in MESA Hispanics (λ = 1.019), WHI Hispanics (λ = 1.158), and WHI African Americans (λ = 1.662). After systematic adjustment for the first two ethnic-specific principal components (PCs)  in linear models, these λ estimates were significantly improved (λ = 1.000 for MESA Hispanics, λ = 1.035 for WHI Hispanics, and λ = 1.034 for WHI African Americans), and all λ values were below our pre-determined threshold of 1.05. Adjustment for additional PCs did not materially alter these estimates. We note that no evidence of genomic inflation was observed in MESA Asians, European Americans, and African Americans. Systematic adjustment for ethnic-specific PC1 and PC2 did not greatly influence the magnitude of the observed p-values in these populations.

### BMI-associated regions

Following SNP quality control (QC) in MESA, 853,278 SNPs in Hispanics, 683,998 in Asians, 871,948 in African Americans, and 749,659 in European Americans were analyzed (Additional file 3: Table S2). The top SNPs (*p* < 5 × 10^−6^) in the MESA ethnic groups are displayed in Table 2.

**Table 2:**
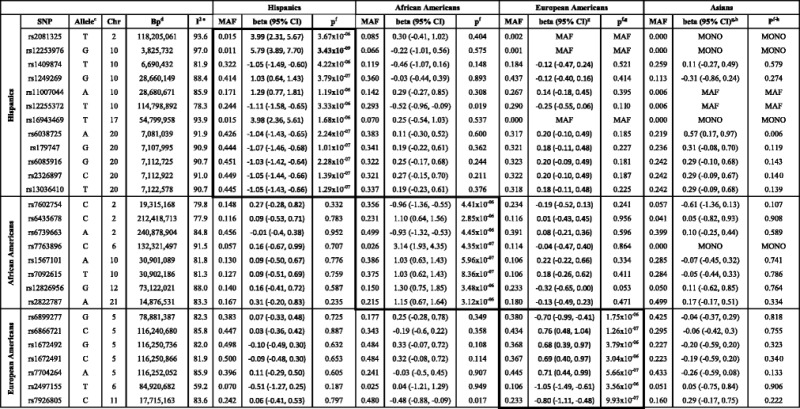
Top SNPs (*p* < 5 × 10^−6^) across the MESA ethnic groups ^a,b^

*Abbreviations*: *Chr* Chromosome, *Bp* base pair, *CI* confidence interval, *MAF* minor allele frequency

^a^ No SNP in Asians with *p* < 5 × 10^−6^

^b^ Boxed values indicate the top SNPs within each ethnic group; p-value in bold indicates genome-wide significant result

^c^ Minor allele

^d^ Base pair positions reported refer to the March 2006 human genome reference assembly (NCBI36/hg18)

^e^ I^2^ values from cross-ethnic meta-analysis

^f^ p-values adjusted for first two principal components and all covariates in ethnic-specific linear regression models

^g^ MAF indicates SNP had a MAF < 0.01 in that particular ethnic group

^h^ MONO indicates SNP is monomorphic in that particular ethnic group

In linear regression analyses, adjusted for all covariates and assuming an additive mode of inheritance, the most significant SNP in Hispanics was rs12253976 (10p15.1) ~8 kb upstream of *KLF6* (beta = 5.792 kg/m^2^ per-allele, 95 % confidence interval (CI): 3.885, 7.698; *p* = 3.43 × 10^−9^). This SNP was the only variant in ethnic-specific analyses to achieve genome-wide significance after Bonferroni adjustment for multiple comparisons. In African Americans, the most significant SNP was rs7763896 (6q23.2) ~7 kb upstream of *CTGF* (beta = 3.140 kg/m^2^ per-allele, 95 % CI: 1.927, 4.352; *p* = 4.35 × 10^−7^). In European Americans, the most significant SNP was rs6866721 (5q23.1; intergenic) near *SEMA6A* (beta = 0.758 kg/m^2^ per-allele, 95 % CI: 0.478, 1.039; *p* = 1.26 × 10^−7^). No SNP in Asians achieved *p* < 5 × 10^−6^. The strength of associations and estimated per-allele effect sizes for these SNPs were relatively consistent across the unadjusted, minimally-adjusted, and fully-adjusted models (Additional file 4: Table S3).

Regional plots visualizing association results for rs12253976, rs7763896, and rs6866721 and their respective flanking region (±500 kb) SNPs are shown in Additional file 5: Figures S3-S5. The chromosome 5 region of European American subjects contains SNPs with low p-values (*p* < 10^−5^) and in strong linkage disequilibrium (LD) (*r*^2^ > 0.8) with rs6866721; these include SNPs rs1672492, rs1672491, and rs7704264 (Table 2). In contrast, the plots for rs7763896 in African Americans and rs12253976 in Hispanics did not show evidence of association for SNPs in their respective flanking regions.

### Ethnic-specificity

The associations between the top SNPs and BMI were generally ethnic-specific (Table 2). Two exceptions were Hispanic SNP rs12255372 and European American SNP rs7926805, whose associations with lower BMI achieved nominal significance (*p* < 0.05) in African Americans. Nevertheless, the I^2^ statistics (from concurrent cross-ethnic meta-analysis in MESA) for all top SNPs were > 50 %, indicating substantial cross-ethnic heterogeneity. Moreover, regional association plots showed that no variants in the vicinity of those SNPs were significantly associated with BMI in the other ethnicities.

### Replication analyses

Additional file 6: Tables S4a-b describes the WHI samples, and Additional file 7: Tables S5a-b shows unadjusted associations between the examined subject characteristics and BMI.

The results of replication analyses in WHI Hispanics and African Americans are shown in Table 3. Three SNPs—rs12255372 (10q25.2; *TCF7L2*)*,* rs6435678 (2q.34; *ERBB4*), and rs6739663 (2q37.3; intergenic)—approached nominal significance in WHI, with betas in the same direction as in MESA. *TCF7L2* and *ERBB4* were associated with BMI in a recent large-scale GWAS meta-analysis [8]. Therefore, we examined these loci more thoroughly.

**Table 3:** Top Hispanic and African American SNPs (*p* < 5 × 10^−6^) across MESA and WHI ^a^

					MESA Hispanics				WHI Hispanics			
SNP	Minor Allele	Chr	Gene	Bp	MAF	beta	95 % CI	p^b^	MAF	beta	95 % CI	p^b^
rs2081325	T	2	intergenic	118,205,061	0.015	3.987	(2.307, 5.667)	3.67 × 10^−06^	0.009	0.155	(−1.174, 1.483)	0.820
rs12253976	G	10	*KLF6*	3,825,732	0.011	5.792	(3.885, 7.698)	3.43 × 10^−09^	0.008	0.598	(−0.837, 2.032)	0.414
rs1409874	T	10	intergenic	6,690,432	0.322	−1.045	(−1.488, −0.602)	4.22 × 10^−06^	0.312	−0.089	(−0.380, 0.202)	0.550
rs1249269	G	10	intergenic	28,660,149	0.414	1.030	(0.635, 1.425)	3.79 × 10^−07^	0.410	−0.197	(−0.472, 0.077)	0.159
rs11007044	A	10	intergenic	28,680,671	0.171	1.290	(0.772, 1.808)	1.19 × 10^−06^	0.176	−0.113	(−0.465, 0.239)	0.530
**rs12255372**	T	10	*TCF7L2*	114,798,892	0.244	−1.111	(−1.578, −0.645)	3.33 × 10^−06^	0.249	−0.304	(−0.613, 0.006)	0.054
rs16943469	T	17	*YPEL2*	54,799,958	0.015	3.984	(2.362, 5.607)	1.68 × 10^−06^	0.006	0.229	(−1.475, 1.932)	0.793
rs6038725	A	20	intergenic	7,081,039	0.426	−1.038	(−1.429, −0.647)	2.24 × 10^−07^	0.384	0.218	(−0.054, 0.489)	0.116
rs179747	G	20	intergenic	7,107,995	0.444	−1.065	(−1.455, −0.676)	1.01 × 10^−07^	0.392	0.228	(−0.044, 0.501)	0.101
rs6085916	G	20	intergenic	7,112,725	0.451	−1.028	(−1.416, −0.641)	2.28 × 10^−07^	0.399	0.197	(−0.078, 0.472)	0.160
rs2326897	C	20	intergenic	7,112,922	0.449	−1.051	(−1.439, −0.662)	1.39 × 10^−07^	0.397	0.197	(−0.073, 0.467)	0.153
rs13036410	T	20	intergenic	7,122,578	0.445	−1.047	(−1.433, −0.661)	1.29 × 10^−07^	0.391	0.219	(−0.050, 0.488)	0.111
					MESA African Americans				WHI African Americans			
SNP	Minor Allele	Chr	Gene	Bp	MAF	beta	95 % CI	p^b^	MAF	beta	95 % CI	p^b^
rs7602754	C	2	intergenic	19,315,168	0.356	−0.956	(−1.362, −0.549)	4.41 × 10^−06^	0.340	0.099	(−0.117, 0.315)	0.370
**rs6435678**	C	2	*ERBB4*	212,418,713	0.231	1.104	(0.643, 1.564)	2.85 × 10^−06^	0.235	0.232	(−0.009, 0.476)	0.061
**rs6739663**	A	2	intergenic	240,878,904	0.499	−0.926	(−1.319, −0.532)	4.45 × 10^−06^	0.494	−0.194	(−0.399, 0.008)	0.062
rs7763896	C	6	*CTGF*	132,321,497	0.026	3.140	(1.927, 4.352)	4.35 × 10^−07^	0.026	−0.175	(−0.806, 0.460)	0.589
rs1567101	A	10	intergenic	30,901,089	0.386	1.030	(0.627, 1.433)	5.96 × 10^−07^	0.392	0.033	(−0.178, 0.242)	0.761
rs7092615	T	10	intergenic	30,902,186	0.375	1.025	(0.619, 1.431)	8.36 × 10^−07^	0.386	0.061	(−0.149, 0.271)	0.567
rs12826956	G	12	intergenic	73,122,021	0.150	1.300	(0.753, 1.847)	3.48 × 10^−06^	0.152	0.010	(−0.277, 0.292)	0.945
rs2822787	A	21	*SAMSN1*	14,876,531	0.215	1.153	(0.670, 1.636)	3.12 × 10^−06^	0.202	−0.067	(−0.318, 0.186)	0.601

*Abbreviations*: *Chr* Chromosome, *Bp* base pair, *CI* confidence interval, *MAF *minor allele frequency

^a^ Bolded SNPs have beta in the same direction and suggestive p-values in MESA and WHI

^b^ p-values adjusted for all ethnic-specific linear regression model covariates; only the MESA analyses are sex-adjusted

### *TCF7L2*

rs12255372 is the Hispanic SNP with most suggestive evidence of replication in WHI (*p* = 3.33 × 10^−6^ in MESA, and *p* = 0.037 and *p* = 0.054 respectively in the WHI age-adjusted and fully-adjusted models; Table 4). Minor allele frequencies (MAFs) for rs12255372 were similar across both Hispanic populations (0.244 in MESA and 0.249 in WHI; Table 3 and Additional file 8: Table S6).

**Table 4:** Association with rs12255372 across the MESA and WHI Hispanics linear regression models

	MESA Hispanics		WHI Hispanics	
	beta (95 % CI)	p	beta (95 % CI)	p
Unadjusted model	−1.144 (−1.623, −0.666)	3.09 × 10^−6^	−0.409 (−0.718, −0.101)	0.009
+ PC 1 and 2	−1.034 (−1.519, −0.548)	3.24 × 10^−5^	−0.342 (−0.655, −0.028)	0.033
+ age and sex^a^	−1.014 (−1.494, −0.534)	3.75 × 10^−5^	−0.334 (−0.647, −0.021)	0.037
Full model^a,b^	−1.111 (−1.578, −0.645)	3.33 × 10^−6^	−0.304 (−0.613, 0.006)	0.054

*Abbreviations*: *CI* confidence interval, *PC* principal components

^a^ Only the MESA analyses are sex-adjusted

^b^ Full model includes all ethnic-specific model covariates

**Fig. 1:**
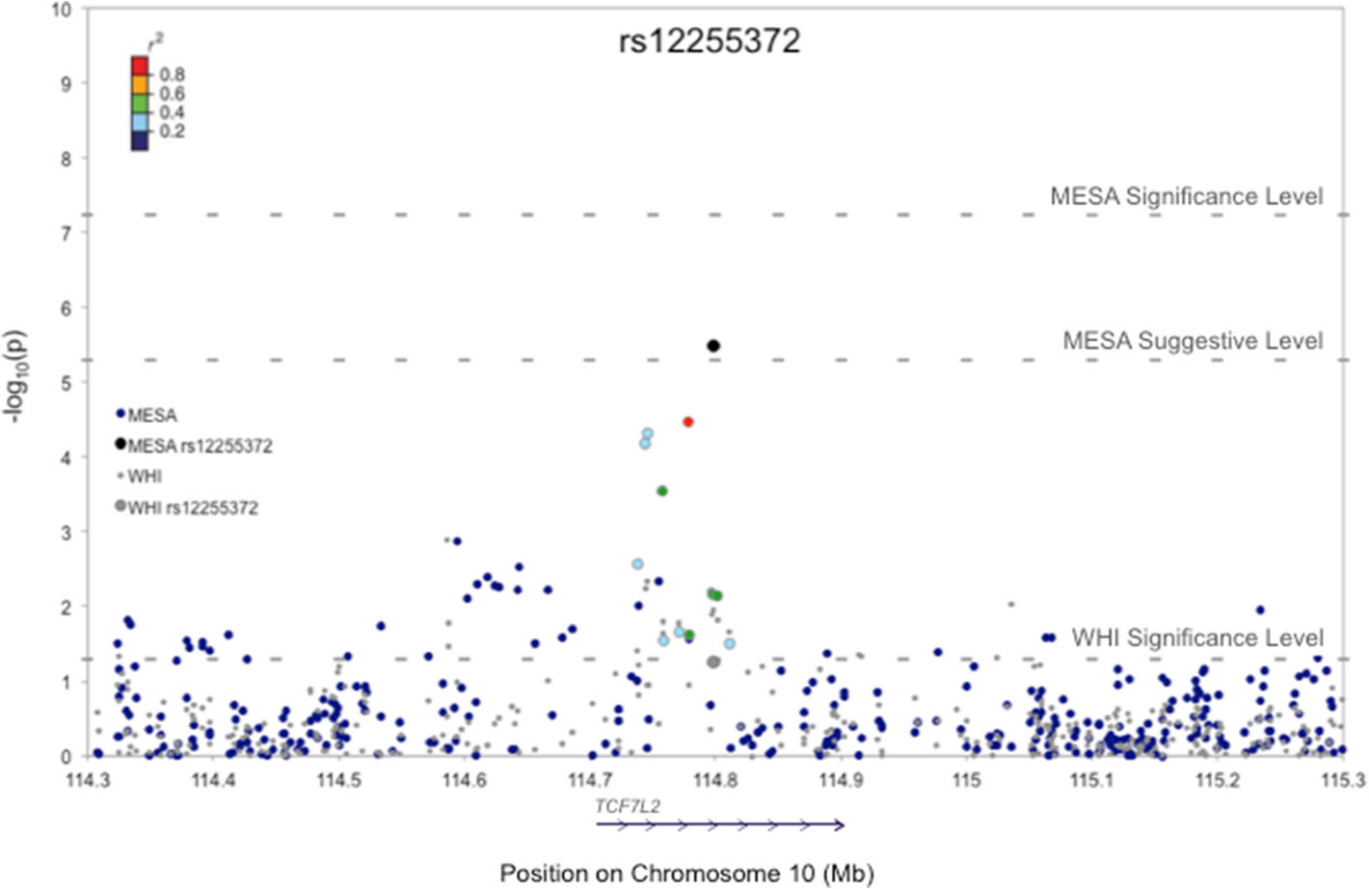
Regional plot for rs12255372 and flanking region (±500 kb) markers in MESA and WHI Hispanics. The associations between BMI and markers (that met our QC metrics) in this region were assessed using linear regression in PLINK. p-values shown were adjusted for the following covariates in MESA and WHI: age, sex, smoking, diabetes, and arthritis. Only MESA analyses were sex-adjusted. Associations were evaluated against a Bonferroni-corrected significance threshold of 5.86 × 10^−8^ in MESA and against a nominal significance threshold of 0.05 in WHI. BMI-associated variants in both populations lie in the intronic region of *TCF7L2*. The blue arrow along the horizontal axis denotes the gene position and direction of transcription.

Figure 1 is a regional plot visualizing association results for rs12255372 and its flanking region (±500 kb) markers in both Hispanic populations. This plots shows four other SNPs—rs7901695, rs4506565, rs4132670, and rs12243326—with low p-values (*p* < 10^−3^) in MESA. These SNPs approached or achieved nominal significance in WHI and had betas of similar magnitude and direction in both Hispanic populations (Table 5). However, only rs12243326 is in strong LD with rs12255372 in our Hispanic populations (*r*^2^ = 0.89 in both MESA and WHI). The other three (rs7901695, rs4506565, and rs4132670) have *r*^2^ values of 0.37-0.54 in MESA and 0.54-0.63 in WHI Hispanics.

**Table 5:** BMI-associated SNPs in *TCF7L2* in MESA and WHI Hispanics

			MESA Hispanics				WHI Hispanics			
			Full model^a^		Full model^a^ w/o diabetes		Full Model^a^		Full model^a^ w/o diabetes	
SNP	Bp	Minor Allele	beta (95 % CI)	p	beta (95 % CI)	p	beta (95 % CI)	p	beta (95 % CI)	p
rs7901695	114,744,078	C	−0.867 (−1.291, −0.442)	6.60 × 10^−05^	−0.812 (−1.244, −0.380)	2.41 × 10^−04^	−0.403 (−0.690, −0.116)	0.006	−0.381 (−0.669, −0.092)	0.010
rs4506565	114,746,031	A	−0.885 (−1.310, −0.460)	4.81 × 10^−05^	−0.835 (−1.267, −0.402)	1.64 × 10^−04^	−0.415 (−0.703, −0.128)	0.005	−0.396 (−0.686, −0.107)	0.007
rs4132670	114,757,761	A	−0.816 (−1.256, −0.377)	2.86 × 10^−04^	−0.793 (−1.241, −0.345)	0.001	−0.337 (−0.628, −0.047)	0.023	−0.321 (−0.614, −0.028)	0.032
rs12243326	114,778,805	G	−0.990 (−1.457, −0.523)	3.46 × 10^−05^	−0.991 (−1.467, −0.515)	4.72 × 10^−05^	−0.251 (−0.560, 0.059)	0.112	−0.243 (−0.554, 0.069)	0.127
rs12255372	114,798,892	T	−1.111 (−1.578, −0.645)	3.33 × 10^−06^	−1.103 (−1.578, −0.628)	5.82 × 10^−06^	−0.304 (−0.613, 0.006)	0.054	−0.296 (−0.608, 0.015)	0.063

*Abbreviations* Bp = base pair, CI = Confidence Interval; w/o = without

^a^ Full model includes: PC1 and PC2, age, sex, and ethnic-specific model covariates; only MESA analyses are sex-adjusted

### *ERBB4*

rs6435678 is the African American SNP with most suggestive evidence of replication in WHI (*p* = 2.85 × 10^−6^ in MESA, and *p* = 0.051 and *p* = 0.061 respectively in the WHI age-adjusted and fully-adjusted models; Table 6). MAFs for rs6435678 were similar across both African American populations (0.231 in MESA and 0.235 in WHI; Table 3 and Additional file 9: Table S7).

**Table 6:** BMI-associated SNPs in *ERBB4* in MESA and WHI African Americans

				MESA African Americans		WHI African Americans	
SNP	Bp	Minor Allele	Model	beta (95 % CI)	p	beta (95 % CI)	p
rs6435678	212,418,713	C	Unadjusted	1.106 (0.647, 1.565)	2.58 × 10^−06^	0.350 (0.098, 0.602)	0.006
			+ PC 1 and 2	1.012 (0.526, 1.498)	4.71 × 10^−05^	0.238 (−0.014, 0.489)	0.064
			+ age and sex^a^	1.121 (0.652, 1.591)	3.10 × 10^−06^	0.249 (−0.001, 0.500)	0.051
			Full model^a,b^	1.104 (0.643, 1.564)	2.85 × 10^−06^	0.232 (−0.009, 0.476)	0.061
rs16847102	212,431,233	A	Unadjusted	0.938 (0.458, 1.418)	1.33 × 10^−04^	0.349 (0.100, 0.599)	0.006
			+ PC 1 and 2	0.911 (0.430, 1.393)	2.15 × 10^−04^	0.238 (−0.012, 0.487)	0.062
			+ age and sex^a^	1.041 (0.576, 1.506)	1.25 × 10^−05^	0.245 (−0.003, 0.493)	0.053
			Full model^a,b^	1.026 (0.570,1.483)	1.12 × 10^−05^	0.219 (−0.021, 0.460)	0.074

*Abbreviations*: *Bp* base pair, *CI* Confidence Interval, *PC* principal components

^a^ Only the MESA analyses are sex-adjusted

^b^ Full model includes all ethnic-specific model covariates

**Fig. 2:**
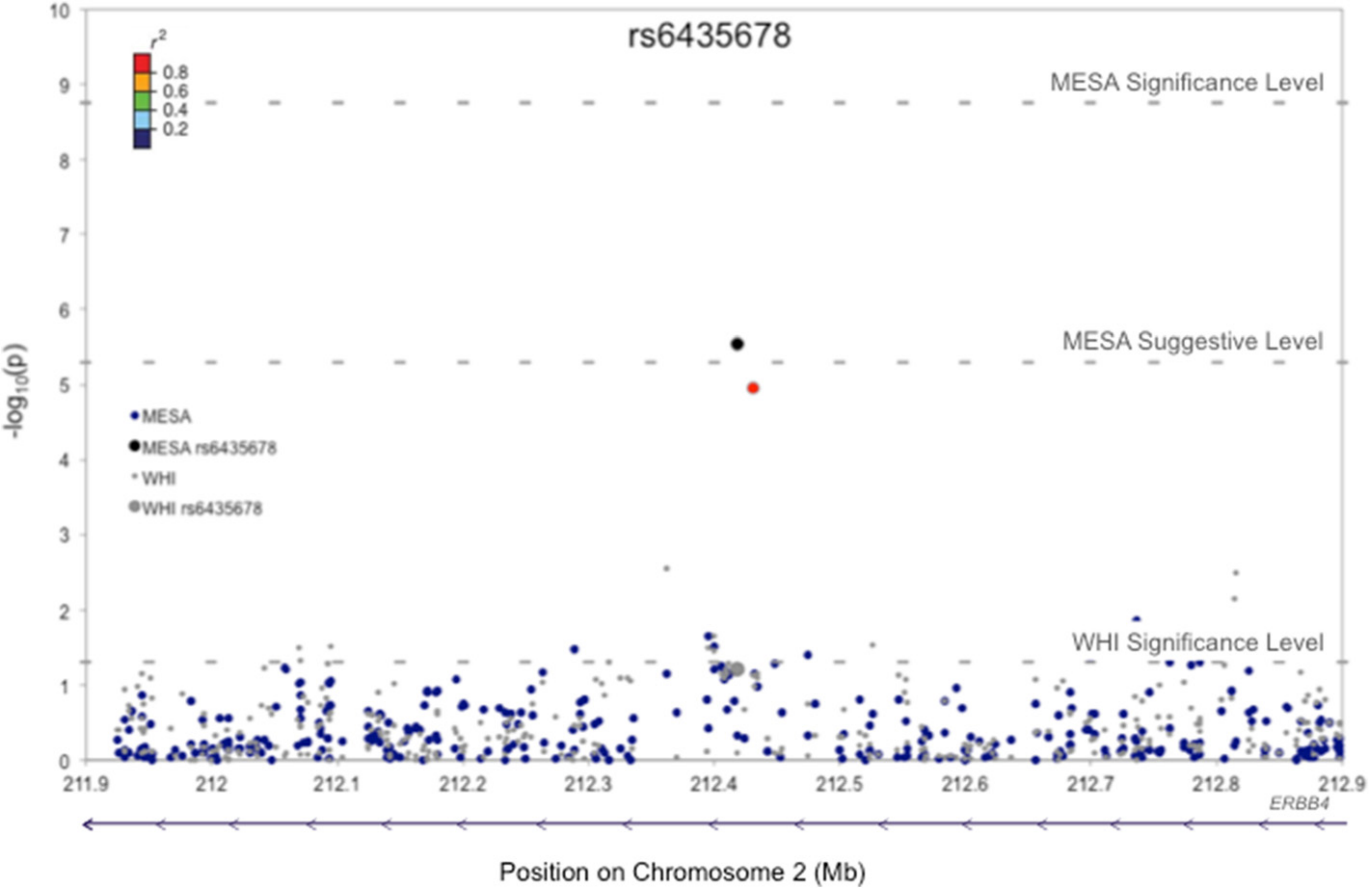
Regional plot for rs6435678 and flanking region (±500 kb) markers in MESA and WHI African Americans. The associations between BMI and markers (that met our QC metrics) in this region were assessed using linear regression in PLINK. p-values shown were adjusted for the following covariates in MESA and WHI: age, sex, education, diabetes, and arthritis. Only MESA analyses were sex-adjusted. Associations were evaluated against a Bonferroni-corrected significance threshold of 5.73 × 10^−8^ in MESA and against a nominal significance threshold of 0.05 in WHI. BMI-associated variants in both populations lie in the intronic region of *ERBB4*. The blue arrow along the horizontal axis denotes the gene position and direction of transcription.

Figure 2 is a regional plot visualizing association results for rs6435678 and its flanking region (±500 kb) markers in both African American populations. This plot shows another BMI-associated SNP at this locus: rs16847102 (*p* = 1.12 × 10^−5^ in MESA, and *p* = 0.053 and *p* = 0.074 respectively in the WHI age-adjusted and fully-adjusted model). rs16847102 is in strong LD with rs6435678 in our African American populations (*r*^2^ = 0.90 and 0.87 respectively in MESA and WHI), and the strength, magnitude, and direction of its BMI-association across both populations mirror that of rs6435678 across the unadjusted, minimally-adjusted, and fully-adjusted models (Table 6).

## Discussion

### Ethnic-specific associations with BMI in MESA

In this study, we investigated genetic associations with BMI via an ethnic-specific GWAS approach. Using data from MESA, we identified suggestive SNPs (*p* < 5 × 10^−6^) displaying ethnic-specific effects on BMI. These include rs12253976 (10p15.1; *KLF6*) in Hispanics, rs7763896 (6q23.2; *CTGF*) in African Americans, and rs6866721 (5q23.1; intergenic) in European Americans. The I^2^ values from concurrent cross-ethnic meta-analyses in MESA provided statistical evidence of substantial cross-ethnic heterogeneity, suggesting that the top SNP effects were not generalizable across ethnicities. Combining results across ethnicities would have masked the SNP effects at these loci (Additional file 10: Table S8). Our findings hence provide support for the hypothesis that ethnic-specific associations are involved in the genetic determination of BMI and highlight the importance of using ethnic-specific approaches for discovery of genetic associations with obesity-related traits.

Ethnic-specificity, which we define as heterogeneity of SNP effects across ethnicities, likely explains some previous failed replications of candidate obesity loci. Examples include an association in the *SIM1* intronic region, discovered in Pima Indians but not generalizable to French Europeans [14], as well as functional coding variant W64R in *ADRB3,* associated with BMI in East Asians but not in Europeans [15]. Ethnic-specificity also has important implications for the evaluation of genetic loci as potential therapeutic agents for obesity: it emphasizes the need for a personalized medicine approach that focuses on identifying the most effective therapies for subjects of different ethnicities.

### Replication in WHI

We sought to replicate the genome-wide significant association for rs12253976 in *KLF6* using data from WHI Hispanics. However, no association signal was detected in this independent population. Since the WHI is an all-female cohort, we explored whether this failed replication could be partially explained by a difference of the SNP effect between men and women. For this purpose, we conducted the following analyses in MESA: a formal test for heterogeneity of the SNP effect by sex [with a cross-product SNP-by-sex interaction term added to the ethnic-specific model] and sex-stratified analyses. As shown in Additional file 11: Table S9, the p-value for the SNP-by-sex interaction for rs12253976 in MESA Hispanics was 2.51 × 10^−4^. While not genome-wide significant, this result prompted us to explore the results of sex-stratified analyses, which revealed that the BMI association signal for rs12253976 in MESA Hispanics was actually stronger in women. Therefore, we concluded that our failure to replicate the findings for this SNP in WHI Hispanics was likely not due to an initial male-driven association in MESA.

We also explored the following explanations for this failed replication: heterogeneity in the BMI distributions of the two Hispanic populations and genetic heterogeneity between the two populations at this locus. As shown in Additional file 12: Figures S6-S7, the BMI distributions of MESA and WHI are similar. Furthermore, the MAFs for this SNP were only slightly different in MESA and WHI (0.011 vs. 0.008; Table 3). However, the genotype distributions were different across the two populations. There were no MESA Hispanic minor allele homozygotes; in fact, the observed association was driven by 27 heterozygotes, who, on average, were 5.8 BMI units heavier than major allele homozygotes (Additional file 13: Table S10). In contrast, all three genotypes were represented in WHI, and the estimated per-allele effect size was more modest. While these differences may be due to sample size differences, they may also indicate true heterogeneity between MESA and WHI. Therefore, the locus containing rs12253976 merits further investigation in other Hispanic populations.

### Association with *TCF7L2*

We also investigated all other suggestive SNPs (*p* < 5 × 10^−6^) in MESA Hispanics in WHI. The Hispanic SNP with most suggestive evidence of replication in WHI was rs12255372, an intronic variant in *TCF7L2*. The regional association plot for this locus displayed four other BMI-associated variants (rs7901695, rs4506565, rs4132670, and rs12243326). Of these, only rs12243326 was in strong LD with rs12255372. The other three SNPs (rs7901695, rs4506565, rs4132670) were in weak LD with rs12255372 in our Hispanic populations, but are expected to be in strong LD with the previously-reported BMI-associated *TCF7L2* variant*,* rs7903146 [8] [estimated *r*^2^ = 0.72-1.00 across the representative International HapMap Project (HapMap phase 3 [16]) populations of Mexican ancestry in Los Angeles, California (MEX), Utah Residents of Northern and Western European Ancestry (CEU), and Chinese in Metropolitan Denver, Colorado (CHD); Additional file 14: Table S11]. Therefore, rs7901695, rs4506565, rs4132670 may serve as proxies for rs7903146 (not genotyped in the Affymetrix 6.0 SNP array), and we conclude that the rs12255372 BMI signal is independent from that of rs7903146. Altogether, this suggests that there are two BMI-associated regions at the *TCF7L2* locus in our Hispanic populations, one tagged by the rs7903146 proxy SNPs and another by rs12255372.

The minor alleles of rs12255372 and rs7903146 have been consistently associated with an increased risk of T2D [17, 18] and have thus been studied extensively in that context. Studies in populations of European ancestry [19], where the two SNPs are in strong LD (Additional file 14: Table S11 and Additional file 15: Figures S8-S13), have proposed rs7903146 as the causal *TCF7L2* variant, given the stronger T2D-association signal at that SNP. However, studies in Hispanic and African American populations [20, 21], where LD between the two SNPs is weak or non-existent (Additional file 14: Table S11 and Additional file 15: Figures S8-S13), have reported association signals at both SNPs, with one study [21] showing that, in Hispanics, rs12255372 yields a stronger T2D-signal than rs7903146. These studies suggest a role for rs12255372 as an independent T2D-signal in *TCF7L2*; and both SNPs may be functionally significant, as both reside in independent, predicted enhancer sites [22].

Our findings suggest a similar story in the context of BMI determination. Locke *et al. *[8], whose most significant *TCF7L2* analysis only included subjects of European ancestry, proposed rs7903146 as a causal variant in this region. Our study, on the other hand, was able to detect two *TCF7L2* signals, since LD does not mask the rs12255372 signal in Hispanic populations.

We note that our study did not find a significant association between the rs7903146 proxy variants (rs7901695, rs4506565, and rs4132670) and BMI in MESA European Americans. We propose two possible explanations for this: that our study was insufficiently powered to detect the purported effect size for rs7903146 in Europeans (−0.02 kg/m^2^ per (minor) allele [8]), and/or that, unlike our study, which consisted entirely of population-based samples, the Locke *et al.* meta-analysis also included case–control studies of T2D. Regarding the latter, Locke *et al*. detected evidence of systematic ascertainment bias at this locus (stronger effects in T2D case-control studies than in population-based studies) [8]. This is in line with candidate gene investigations in population-based samples of European ancestry, such as DESIR [23] and the Framingham Heart Study [24], which refuted prior claims of *TCF7L2* BMI-associations made by studies examining this relationship only among individuals with T2D [25].

Nonetheless, in our Hispanic population-based samples, we find that the minor alleles of *TCF7L2* intronic variants are associated with lower BMI. Since our regression models had adjusted for diabetes, we examined the effect of removing this variable from the models in *ad hoc* analyses. Table 5 shows that the associations with lower BMI at this locus were either attenuated or unchanged after removing this variable. Thus, in our Hispanic populations, *TCF7L2* intronic variants are associated with lower BMI independently of T2D.

### Association with *ERBB4*

We also investigated all other suggestive SNPs (*p* < 5 × 10^−6^) in MESA African Americans in WHI. The SNP with the most suggestive p-values across both African American populations was rs6435678, an intronic variant in *ERBB4*. The regional plot for this locus showed that the association pattern of the rs6435678 flanking region markers reflected the LD structure of the MESA and WHI African American populations, thus lending additional support to our finding. We note that, upon inspecting this region across the other ethnicities, we found no significant evidence of BMI-associations. Thus, our data suggest that the BMI-effect of rs6435678 may be specific to African Americans, though further investigation in independent multiethnic samples is necessary to substantiate this finding.

*ERBB4* was previously linked to BMI in populations of European ancestry via an association with rs7599312 [8], located ~10 kb upstream of this gene. rs6435678, which resides in intron 3, is not in LD with rs7599312 in our African American samples (*r*^2^ = 0.00 and 0.01 respectively in MESA and WHI). Thus, we conclude that rs6435678 represents a novel signal in *ERBB4*. We note that no association with rs7599312 was detected in MESA European Americans. However, this was not surprising because the purported effect size for this SNP is only 0.02 kg/m^2^ per-allele in populations of European ancestry [8], which our study was not powered to detect. We also note that there is no LD between rs7599312 and rs6435678 in European Americans (*r*^2^ = 0.00 in MESA).

An association between variants in *ERBB4* and BMI is biologically plausible. *ERBB4* encodes a receptor tyrosine kinase expressed in various tissues, including liver and pancreas. In the liver, ERBB4 regulates lipogenesis by binding to Neuregulin 4, an epidermal growth factor secreted by brown adipose tissue [26]. In the pancreas, ERBB4 is involved in the epidermal growth factor receptor signaling pathway, which regulates islet cell differentiation [27, 28] and β-cell signal transduction, and whose disruption has been linked to impaired glucose tolerance and reduced insulin response in mice [29].

### Strengths and limitations

Conducting our GWAS analyses within MESA gave us the unique opportunity to compare genetic associations across four ethnic groups that were sampled in the same fashion from the same underlying source population. However, we note that stratifying MESA by ethnicity limited our statistical power to detect variants with small effect sizes: for variants with MAF ≥ 0.2, we had ≥ 80 % power to detect effect sizes of ≥ 1.4, ≥ 1.7, ≥ 1.2, and ≥ 1.7 kg/m^2^ in Asians, Hispanics, European Americans, and African Americans, respectively (Additional file 16: Tables S12). This could explain why only one SNP achieved genome-wide significance; why no suggestive SNPs (*p* < 5 × 10^−6^) were identified in Asians; or why *FTO* was only nominally associated in European Americans and had inconsistent effects in other ethnicities. We acknowledge that a fixed-effects meta-analysis of ethnic-specific GWAS data would be better powered than our ethnic-stratified approach. However, in our own meta-analysis in MESA, we detected substantial evidence of cross-ethnic heterogeneity. Thus, we concluded that pooled effect estimates across ethnicities should not be presented; they would be meaningless, since the effect of the SNPs is not common to all ethnicities.

Another limitation is that many of the SNPs that we identified in MESA did not show evidence of replication in WHI. The WHI Hispanic and African American populations—though similar to their MESA counterparts with respect to BMI distribution (Additional file 12: Figures S6-S7) and MAFs at the evaluated loci (Table 3)—are composed entirely of women, and this had the potential to affect our ability to replicate our findings. Given the results of our formal tests of heterogeneity of the SNP effects by sex and the accompanying sex-stratified analyses in MESA (Additional file 11: Table S9), there is no evidence to suggest that failure to replicate our findings is due to initial male-driven associations in MESA.

A final limitation is that the genotyping platform used by the MESA and WHI studies (the Affymetrix 6.0 SNP array) was designed to optimize coverage of common genetic variants (MAF ≥ 0.1). As noted in [30], lower frequency variants are more likely to be ethnic-specific. Therefore, in multiethnic studies, the use of custom arrays optimized for minority populations would be optimal.

## Conclusions

By employing an ethnic-specific GWAS approach, we identified suggestive BMI-associated SNPs in Hispanics, African Americans, and European Americans that can be explored in future studies. The Hispanic and African American SNPs directed us to *TCF7L2* and *ERBB4*. We show that the *TCF7L2* intronic region contains two BMI-association signals in Hispanics, one of which (rs12255372) would have likely gone undetected had we not employed an ethnic-specific analytic approach. We also show that the *ERBB4* intronic region contains a novel BMI association signal (rs6435678) that may be specific to African Americans.

Overall, our data suggest that ethnic-specific associations are involved in the genetic determination of BMI. The existence of heterogeneous SNP effects across ethnicities highlights the need for utilizing ethnic-specific approaches for discovery of genetic associations and may have important implications for the development of gene-based therapies for common diseases such as obesity.

## Methods

### Discovery phase

Subjects providing data for the discovery phase included 1235 Hispanic, 706 Asian, 1549 African American, and 2395 European American subjects recruited into MESA, a multi-center, prospective study of risk factors affecting CVD progression. Recruitment has been described elsewhere [31]. Briefly, 6814 men and women aged 45–84 years were recruited from six U.S. field centers in 2000–2002. MESA ascertained subject race and ethnicity via a standard questionnaire that adopted the definitions used by the U.S. Office of Management and Budget (OMB). [For simplicity, our present study uses the term ‘ethnicity’ to refer to the four racial-ethnic groups defined in MESA]. MESA recruited overlapping ethnic groups among field centers to minimize confounding by ethnicity by site [31]. Blood was collected from each subject, and DNA samples were genotyped for 909,622 SNPs using the Affymetrix 6.0 SNP array. Samples were required to have a call rate > 95 %. Further details of sample preparation and genotyping are described elsewhere [31].

Genotype and phenotype information for MESA were obtained from the National Center for Biotechnology Information's database of Genotypes and Phenotypes (NCBI dbGaP study accession: phs000209.v11.p3 MESA SNP Health Association Resource (SHARe)). For the present analysis, MESA was stratified into four ethnic-specific samples. EIGENSTRAT [32] analyses verified that the MESA ethnic groups were clustering together based on genotype data (Additional file 17: Figures S14-S15). Subjects analyzed met the QC thresholds described below and summarized in Additional file 3: Table S2 and had complete data for all ethnic-specific model covariates.

All phenotypic data used herein were obtained at the MESA baseline examination. The primary outcome variable was BMI (kg/m^2^), calculated from height and weight measurements collected by trained staff at the field centers. All genotyped subjects had baseline BMI data. Variables examined as potential covariates due to their previously-reported associations with BMI were sex, baseline age, education, income, smoking, arthritis, diabetes, and physical activity. Details regarding covariate measurement are provided in Additional file 18: Supplemental Methods.

Ethnic-specific associations between BMI and potential covariates were examined in SAS 9.3 (SAS Institute, Cary NC). Ethnic-specific linear models were then built, with the most parsimonious models selected via backwards elimination of covariates with *p* > 0.05 until model adjusted-r^2^ values were maximized. Age and sex were retained in the models regardless of the statistical significance of their associations with BMI. Individuals with missing values for any covariate included in the final ethnic-specific models were removed from the analyses. Details of variable parameterization and evaluation of the appropriateness of using linear regression on these data are provided in Additional file 18: Supplemental Methods.

Ethnic-specific SNP QC analyses were performed within PLINK [33]. We excluded SNPs based on low call rate (< 98 %), low MAF (< 0.01), and significant deviation from Hardy-Weinberg equilibrium (*p* ≤ 5.5 × 10^−8^) (Additional file 3: Table S2).

Genetic QC procedures also included assessments for cryptic relatedness and population stratification. Cryptic relatedness between subjects in each ethnicity was examined within PLINK using pair-wise identity-by-descent (IBD) estimation. Pairs with $\widehat{\pi}$ (estimated proportion of genome shared IBD) > 0.2 were inspected, and only one subject from each family was included. Population stratification was assessed by calculating genomic inflation factors (λ) in PLINK and conducting ethnic-specific PC analysis in EIGENSTRAT. The first two ethnic-specific PCs were systematically added as covariates to each ethnic-specific linear model; and these PCs were sufficient to control for genomic inflation (all λ below a pre-determined threshold of 1.05).

Associations between individual SNPs and BMI were tested in PLINK using ethnic-specific linear regression analyses, with initial adjustment for the first two PCs; additional adjustment for age and sex; and full adjustment for ethnic-specific covariates. Genome-wide significance was evaluated against ethnic-specific Bonferroni-corrected thresholds, as performed in [34]. The significance thresholds were 5.86 × 10^−8^ (0.05/853,278), 7.31 × 10^−8^ (0.05/683,998), 5.73 × 10^−8^ (0.05/871,948), and 6.67 × 10^−8^ (0.05/749,659) in Hispanics, Asians, African Americans, and European Americans, respectively.

After identifying SNPs with suggestive p-values (*p* < 5 × 10^−6^) in each MESA ethnic group, we evaluated the generalizability of these associations to other ethnic groups. The ±500 kb flanking regions of the top SNPs were examined across all ethnicities to account for potential ethnic differences in LD patterns.

Lastly, we conducted cross-ethnic meta-analyses using an inverse-variance method in PLINK and calculated the I^2^ statistic for each SNP. I^2^ values quantify the percentage of variability in effect estimates attributable to heterogeneity rather than to chance alone [35, 36], and, in this context, they can be interpreted as a measure of cross-ethnic heterogeneity.

### Replication phase

Suggestive SNPs (*p* < 5 × 10^−6^) identified in MESA Hispanics and African Americans were investigated in 3379 Hispanic and 6871 African American women from WHI. Recruitment and selection criteria for WHI have been described previously [37]. Briefly, WHI recruited post-menopausal women aged 50–79 years at 40 U.S. field centers in 1993–1998. WHI also ascertained subject race and ethnicity via a standard questionnaire that adopted OMB definitions. [As for MESA, our present study uses the term ‘ethnicity’ to refer to the racial-ethnic groups defined in WHI]. Of 161,808 subjects recruited, 12,008 were genotyped for the WHI SNP SHARe project (NCBI dbGaP study accession: phs000200.v10.p3) using the Affymetrix 6.0 SNP array. Of these, 3560 Hispanics and 8359 African Americans had baseline BMI data.

All measurements used herein were obtained at the WHI baseline examination. BMI was calculated from height and weight measurements collected by trained staff. Covariates were selected based on ethnic-specific regression models built for MESA Hispanics and African Americans (Additional file 18: Supplemental Methods.).

SNPs and subjects were excluded from genome-wide SNP analyses if they did not meet the QC criteria outlined previously (Additional file 3: Table S2). Replication results were evaluated against a nominal significance level of 0.05 and same direction of effect.

## Abbreviations

Not applicable.

## Additional files

Additional file 1: Table S1. Unadjusted associations between participant characteristics and BMI across the MESA ethnic groups. (XLSX 51 kb) Additional file 2: Figures S1-S2. Quantile-quantile p-value plots for MESA and WHI, pre- and post- adjustment for population stratification. (DOCX 255 kb) Additional file 3: Table S2. Summary of Subject and SNP Quality Control Procedures. (XLSX 10 kb) Additional file 4: Table S3. Top candidate SNP in each MESA ethnic group across all regression models. (XLSX 46 kb) Additional file 5: Figures S3-S5. Regional association plots for top candidate SNPs and flanking region markers (±500 kb) in MESA. (DOCX 257 kb) Additional file 6: Tables S4a-b. Description of the WHI SHARe Hispanic and African American study populations. (XLSX 41 kb) Additional file 7: Tables S5a-b. Unadjusted associations between participant characteristics and BMI in WHI Hispanics and African Americans. (XLSX 44 kb) Additional file 8: Table S6. Average BMI by genotype for rs12255372 in MESA and WHI Hispanics. (XLSX 39 kb) Additional file 9: Table S7. Average BMI by genotype for rs6435678 in MESA and WHI African Americans. (XLSX 40 kb) Additional file 10: Table S8. Meta-analysis for top candidate SNP in MESA Hispanics, African Americans, and European Americans. (XLSX 46 kb) Additional file 11: Table S9. Evaluation of heterogeneity of SNP effects by sex for top MESA Hispanic and African American SNPs (*p* < 5 × 10^−6^). (XLSX 53 kb) Additional file 12: Figures S6-S7. Histograms and quantile-quantile plots for ethnic-specific BMI distributions in MESA and WHI. (DOCX 377 kb) Additional file 13: Table S10. Average BMI by genotype for the top candidate SNP in each MESA ethnic group. (XLSX 41 kb) Additional file 14: Table S11. Summary of association results and LD structure analysis of *TCF7L2* variants. (XLSX 59 kb) Additional file 15: Figures S8-S13. Ethnic-specific LD structures of *TCF7L2* in MESA and WHI. (DOCX 739 kb) Additional file 16: Table S12. Power estimations in MESA and WHI. (XLSX 50 kb) Additional file 17: Figures S14-S15. PC analyses across the four MESA ethnic groups and across the WHI ethnic groups, plotted in the first two dimensions. (DOCX 622 kb) Additional file 18: Supplemental Methods. Details regarding ethnic-specific linear regression model covariates and checks for normality of the response variable. (DOCX 184 kb)
