# Supplementary material for: Multiethnic genome-wide association study identifies ethnic-specific associations with body mass index in Hispanics and African Americans
Source: BMC Genet. 2016 Jun 13;17:78. doi: 10.1186/s12863-016-0387-0 (PMC4907283; doi:10.1186/s12863-016-0387-0)
Supplement: Additional file 2: Figures S1-S2. — Quantile-quantile p-value plots for MESA and WHI, pre- and post- adjustment for population stratification. (DOCX 255 kb) [file 12863_2016_387_MOESM2_ESM.docx]

# Figure S1. QQ p-value plots for MESA, pre- and post- adjustment for population stratification


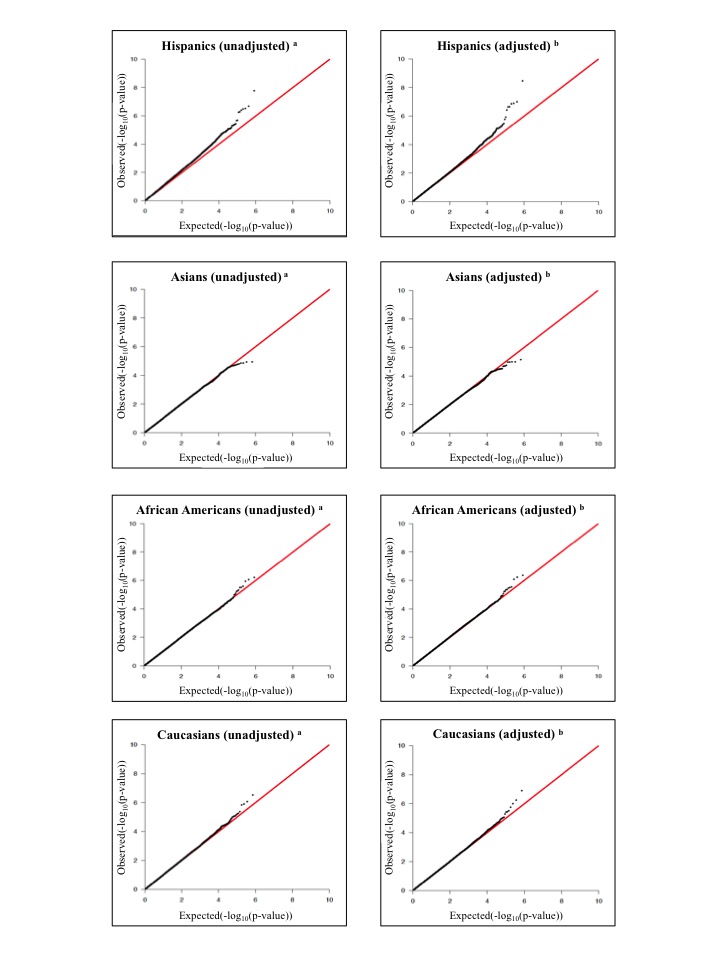


**Abbreviation:** QQ=quantile-quantile

^a^ p-values adjusted only for ethnic-specific model covariates

^b^ p-values adjusted for ethnic-specific model covariates and the first ethnic-specific two principal components

P-values were calculated using linear regression in PLINK[1]. QQ-plots were constructed in R.

# Figure S2. QQ p-value plots for WHI, pre- and post- adjustment for population stratification

^
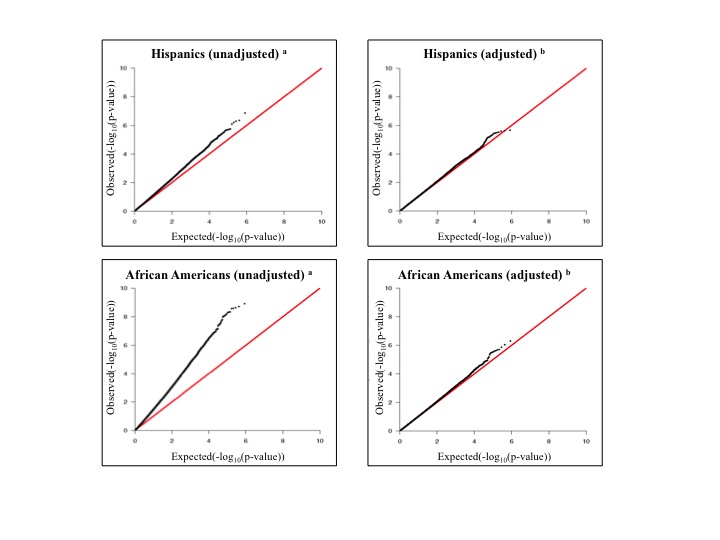
^

**Abbreviation:** QQ= quantile-quantile

^a^ p-values adjusted only for ethnic-specific model covariates

^b^ p-values adjusted for ethnic-specific model covariates and the first two ethnic-specific principal components

P-values were calculated using linear regression in PLINK[1]. QQ-plots were constructed in R.

**Reference.**

1. Purcell, S., et al., *PLINK: a tool set for whole-genome association and population-based linkage analyses.* Am J Hum Genet, 2007. **81**(3): p. 559-75.
