# Supplementary material for: Multiethnic genome-wide association study identifies ethnic-specific associations with body mass index in Hispanics and African Americans
Source: BMC Genet. 2016 Jun 13;17:78. doi: 10.1186/s12863-016-0387-0 (PMC4907283; doi:10.1186/s12863-016-0387-0)
Supplement: Additional file 5: Figures S3-S5. — Regional association plots for top candidate SNPs and flanking region markers (±500 kb) in MESA. (DOCX 257 kb) [file 12863_2016_387_MOESM5_ESM.docx]

# Figure S3. Regional association plot for top candidate SNP rs12253976 and flanking region markers (+ 500kb) in MESA Hispanics


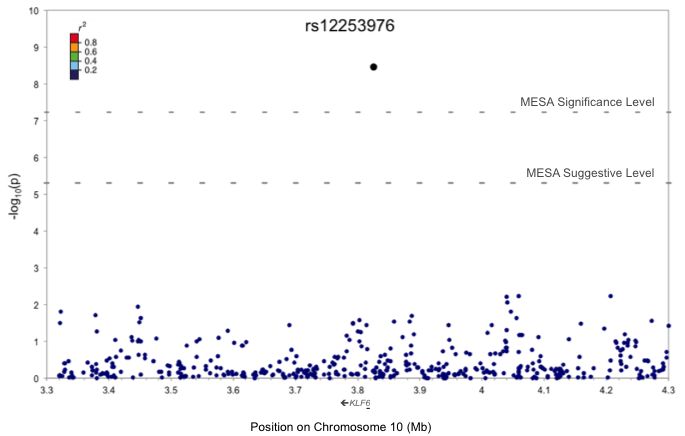


The association between BMI and markers (that met our quality control metrics) in this region was assessed using linear regression in PLINK[1]. p-values shown were adjusted for the following covariates: age, sex, smoking, diabetes, and arthritis. Associations in MESA Hispanics were evaluated against a Bonferroni-corrected p-value of 5.86x10^-8^. The nearest gene to rs12253976 is *KLF6*.

**Figure S4. Regional association plot for top candidate SNP rs7763896 and flanking region markers (**+**500kb) in MESA African Americans**


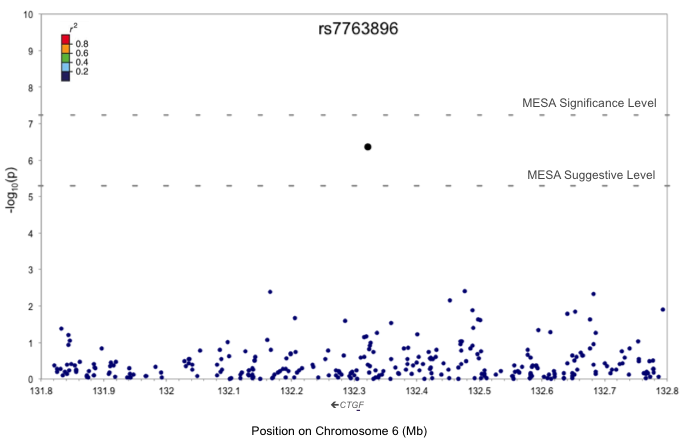


The association between BMI and markers (that met our quality control metrics) in this region was assessed using linear regression in PLINK[6]. p-values shown were adjusted for the following covariates: age, sex, education, diabetes, and arthritis. Associations in MESA African Americans evaluated against a Bonferroni-corrected p-value of 5.73x10^-8^. The nearest gene to rs7763896 is *CTGF*.

#

# Figure S5. Regional association plot for top candidate SNP rs6866721 and flanking region markers (+500kb) in MESA European Americans


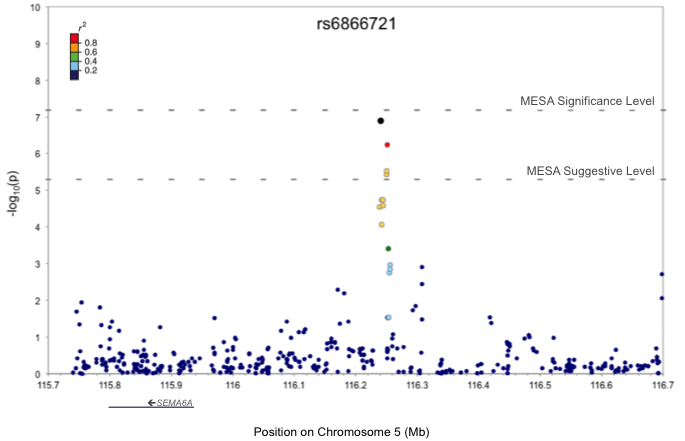


The association between BMI and markers (that met our quality control metrics) in this region was assessed using linear regression in PLINK[1]. p-values shown were adjusted for the following covariates: age, sex, income, education, smoking, physical activity, diabetes and arthritis. Associations in MESA European Americans were evaluated against a Bonferroni-corrected p-value of 6.67x10^-8^. The nearest gene to rs6866721 is *SEMA6A*.

**Reference.**

1. Purcell, S., et al., *PLINK: a tool set for whole-genome association and population-based linkage analyses.* Am J Hum Genet, 2007. **81**(3): p. 559-75.
