# Supplementary material for: Multiethnic genome-wide association study identifies ethnic-specific associations with body mass index in Hispanics and African Americans
Source: BMC Genet. 2016 Jun 13;17:78. doi: 10.1186/s12863-016-0387-0 (PMC4907283; doi:10.1186/s12863-016-0387-0)
Supplement: Additional file 12: Figures S6-S7. — Histograms and quantile-quantile plots for ethnic-specific BMI distributions in MESA and WHI. (DOCX 377 kb) [file 12863_2016_387_MOESM12_ESM.docx]

# Figure S6. Histograms and QQ plots for ethnic-specific BMI distributions in MESA

**MESA African Americans**


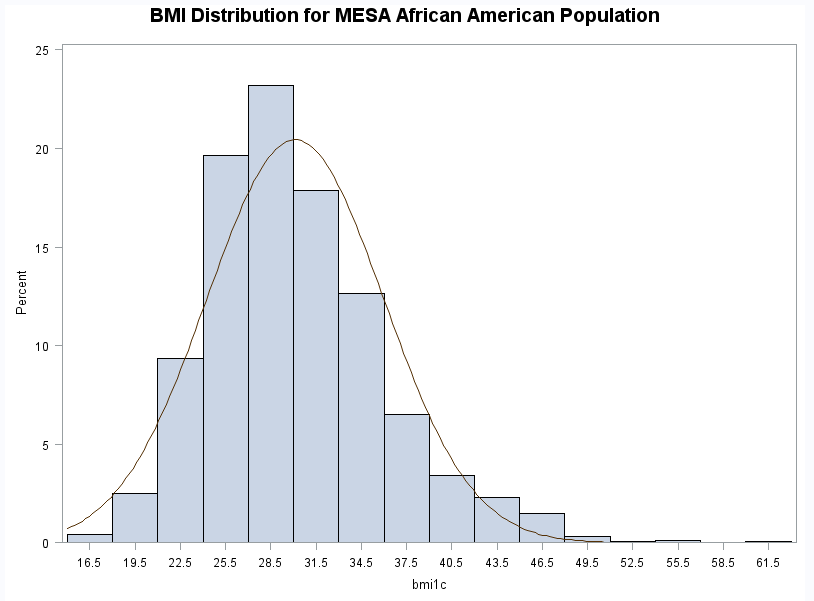

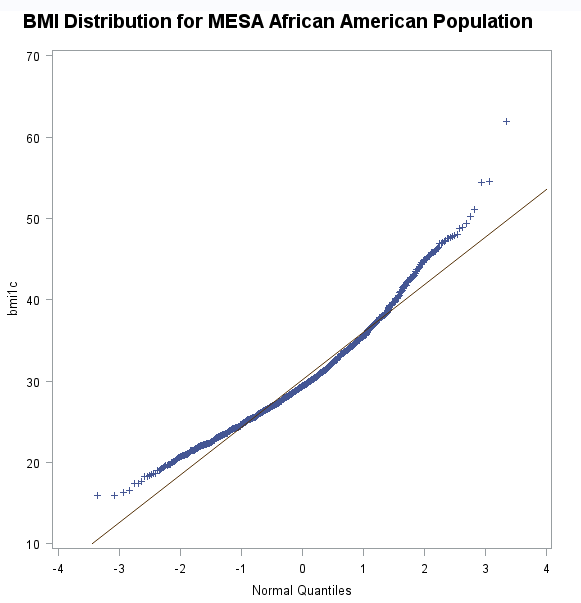


**MESA European Americans**


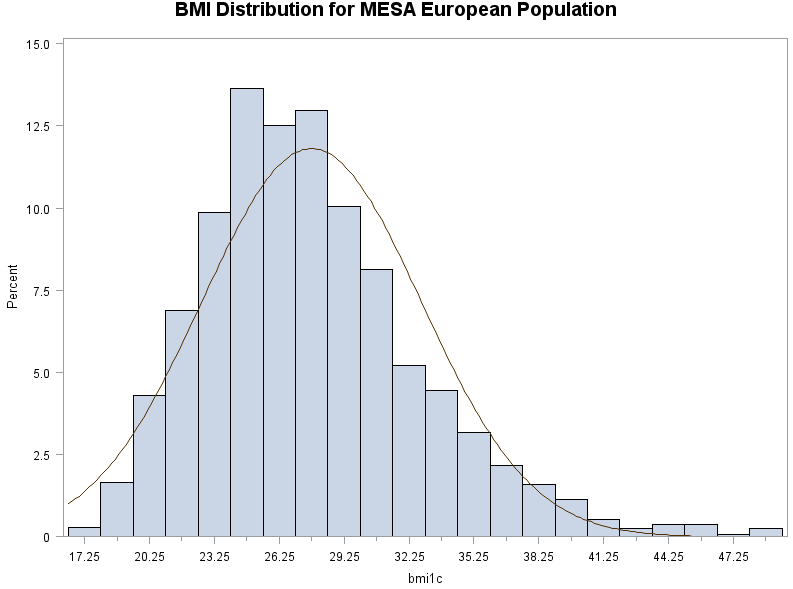

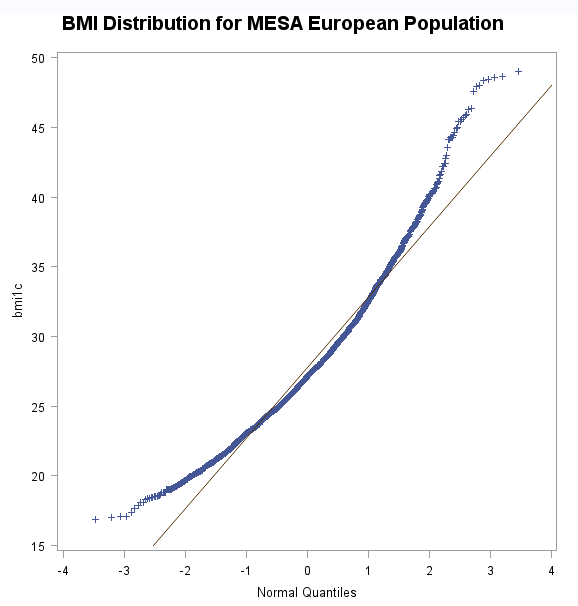


# Figure S6 (continued).

**MESA Hispanics**


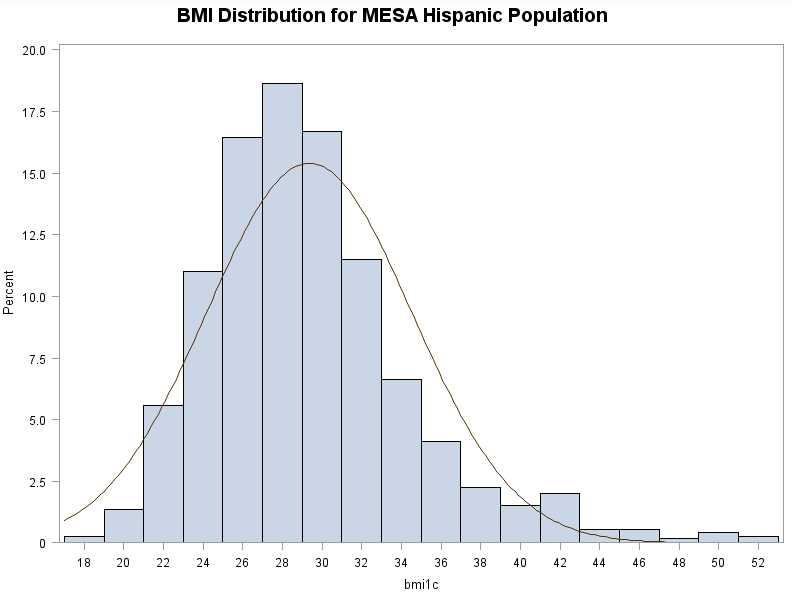

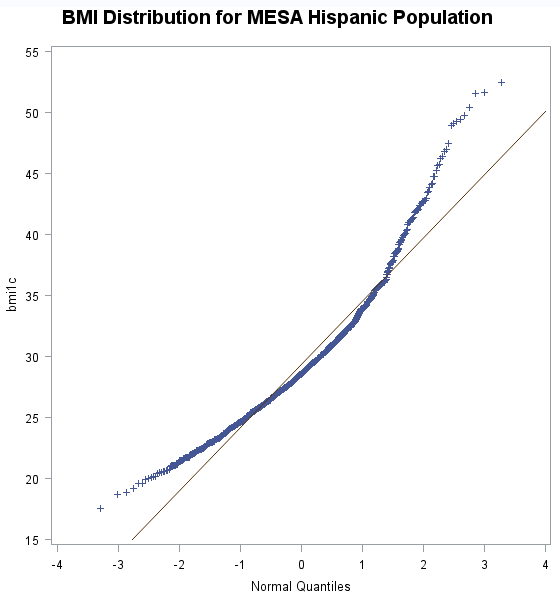


**MESA Asians**


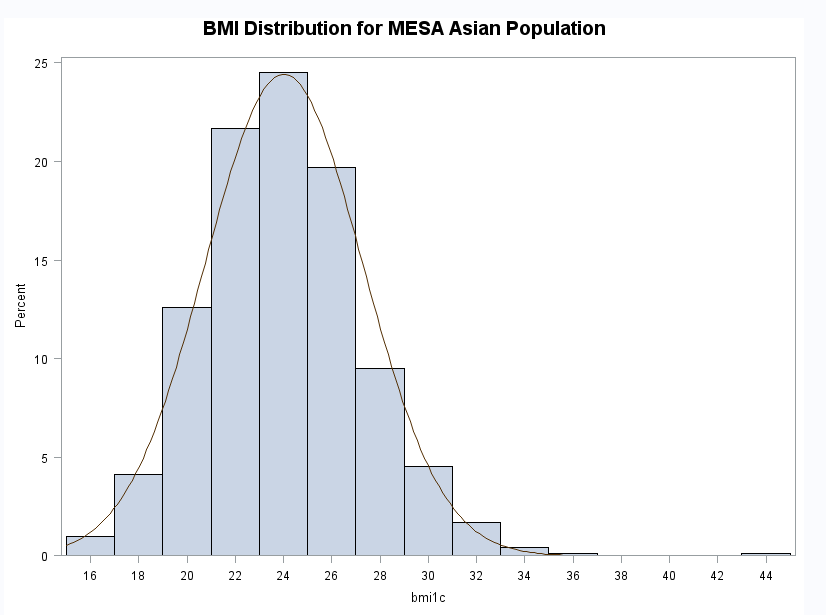

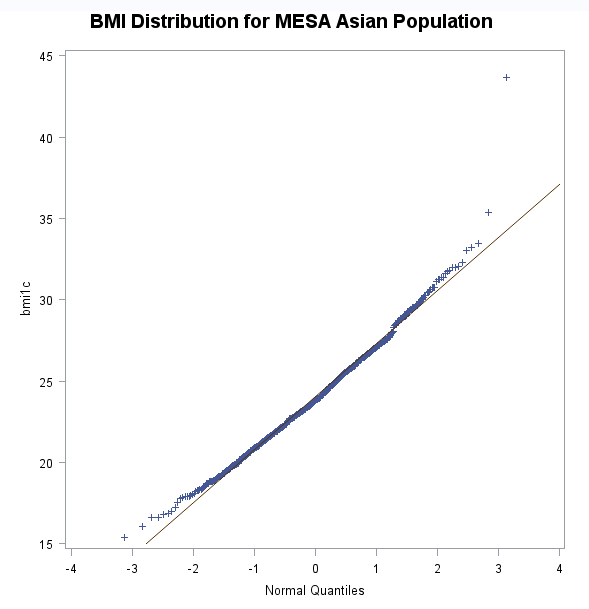


Histograms of the BMI distributions across the four MESA ethnic groups were constructed in SAS 9.3 (SAS Institute, Cary NC). Normal-quantile (QQ) plots were also constructed in SAS as a visual check for the linear regression assumption of normality of error terms (which is usually guaranteed by normality of the response variable). It can be seen that the BMI distributions were right-skewed (to varying degrees) across all ethnicities. In addition, the observed BMI values at the upper tails of the distributions deviated from what is strictly expected under the null hypothesis of normality. However, BMI was not transformed for reasons detailed in the Supplemental Methods.

# Figure S7. Histograms and QQ plots for ethnic-specific BMI distributions in WHI

**WHI Hispanics**


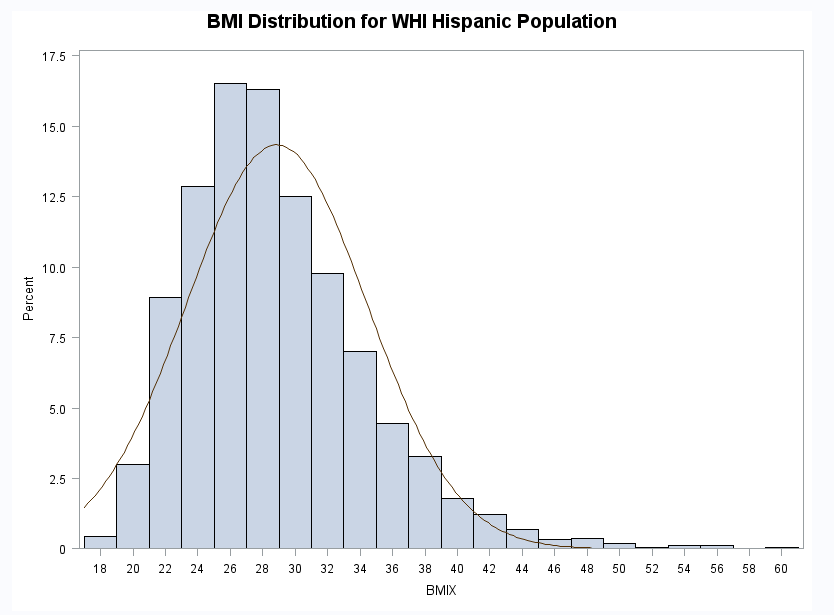

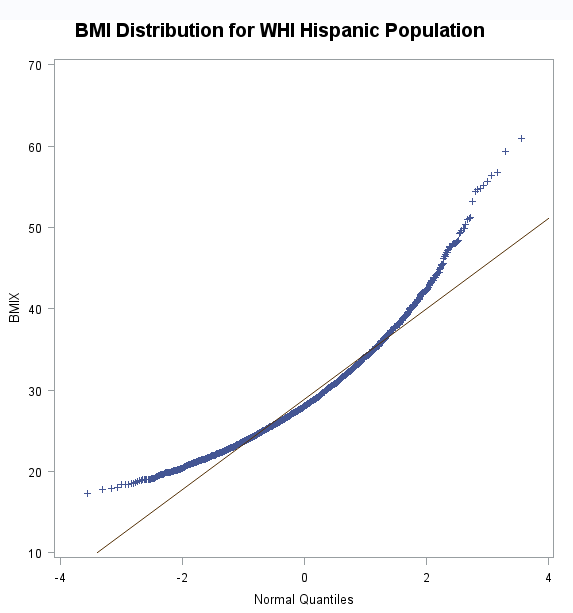


**WHI African Americans**


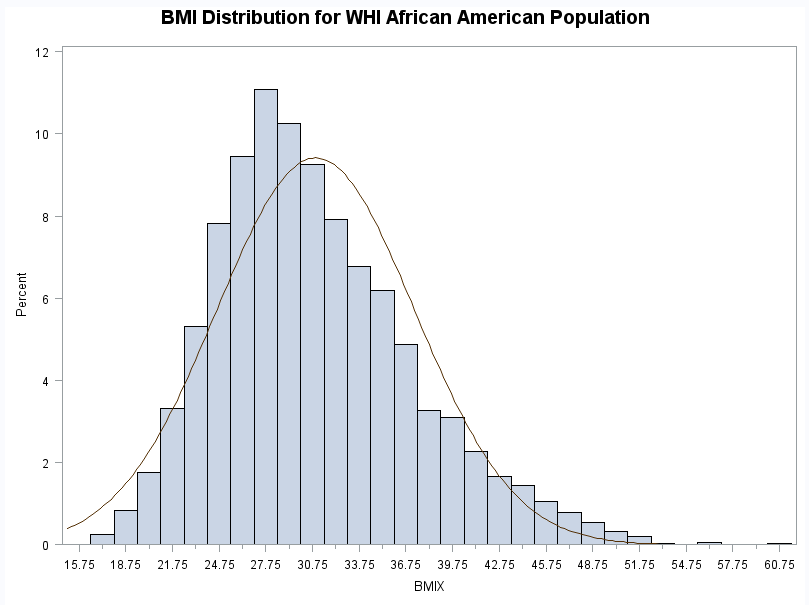

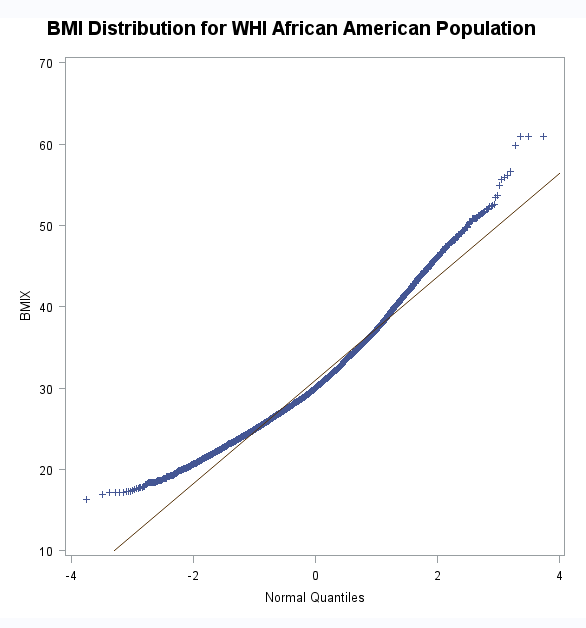


Histograms of the BMI distributions across the two WHI ethnic groups were constructed in SAS 9.3 (SAS Institute, Cary NC). Normal-quantile (QQ) plots were also constructed in SAS as a visual check for the linear regression assumption of normality of error terms (which is usually guaranteed by normality of the response variable). It can be seen that the BMI distributions were right-skewed (to varying degrees) across both ethnic groups. In addition, the observed BMI values at the upper tails of the distributions deviated from what is strictly expected under the null hypothesis of normality. However, BMI was not transformed for reasons detailed in the Supplemental Methods.
