# Supplementary material for: Multiethnic genome-wide association study identifies ethnic-specific associations with body mass index in Hispanics and African Americans
Source: BMC Genet. 2016 Jun 13;17:78. doi: 10.1186/s12863-016-0387-0 (PMC4907283; doi:10.1186/s12863-016-0387-0)
Supplement: Additional file 15: Figures S8-S13. — Ethnic-specific LD structures of TCF7L2 in MESA and WHI. (DOCX 739 kb) [file 12863_2016_387_MOESM15_ESM.docx]

**Figure S8. LD structure of *TCF7L2* in MESA Hispanics ^a,b^**

**
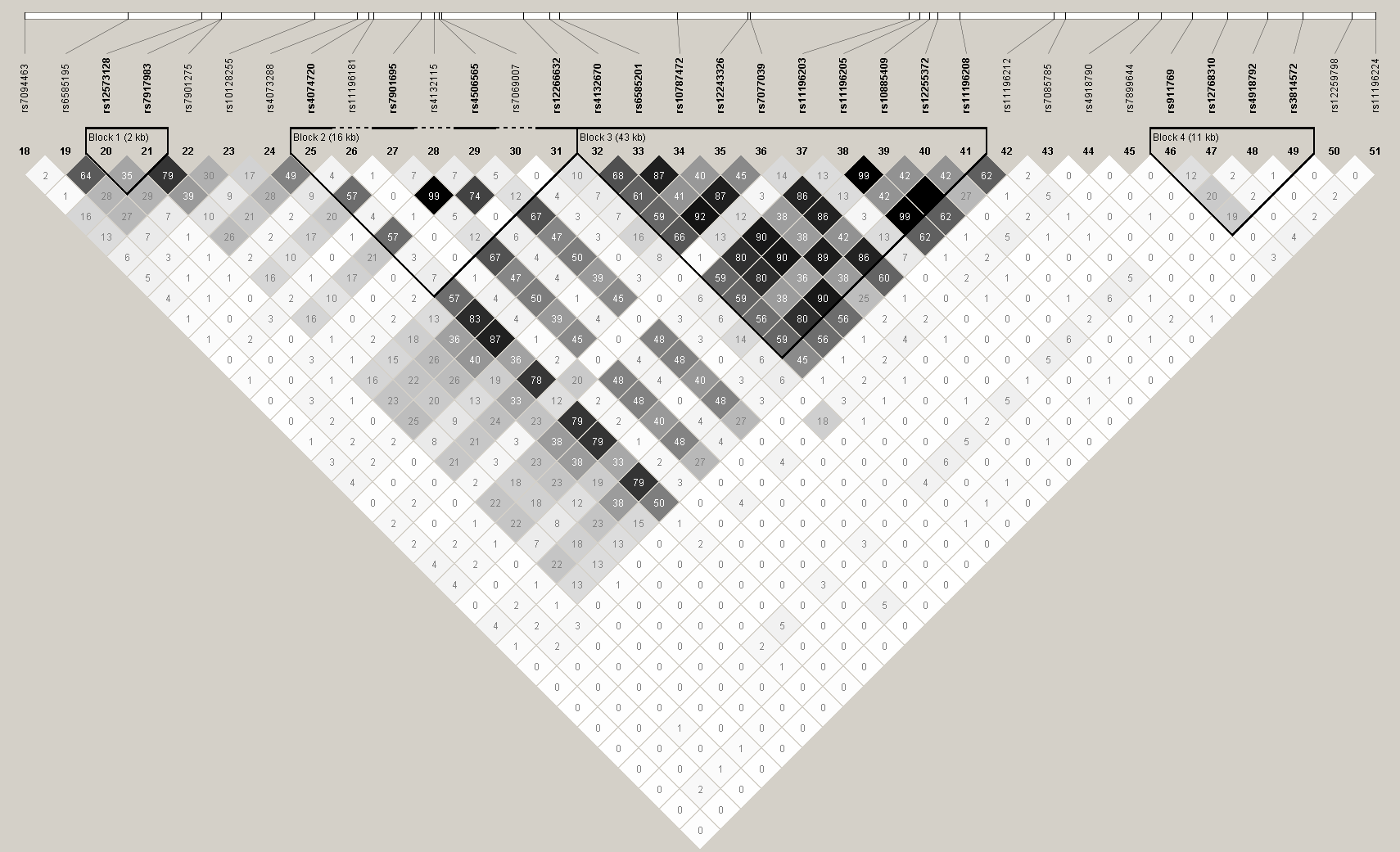
**

^a^ LD estimation performed in HaploView

^b^ Region shown captures the respective ±50kb regions of rs7903146 and rs12255372; markers included are those that passed quality control in ethnic-specific GWAS); rs7903146 not genotyped in Affymetrix 6.0 Platform

**Figure S9. LD structure of *TCF7L2* in MESA African Americans ^a,b^**

**
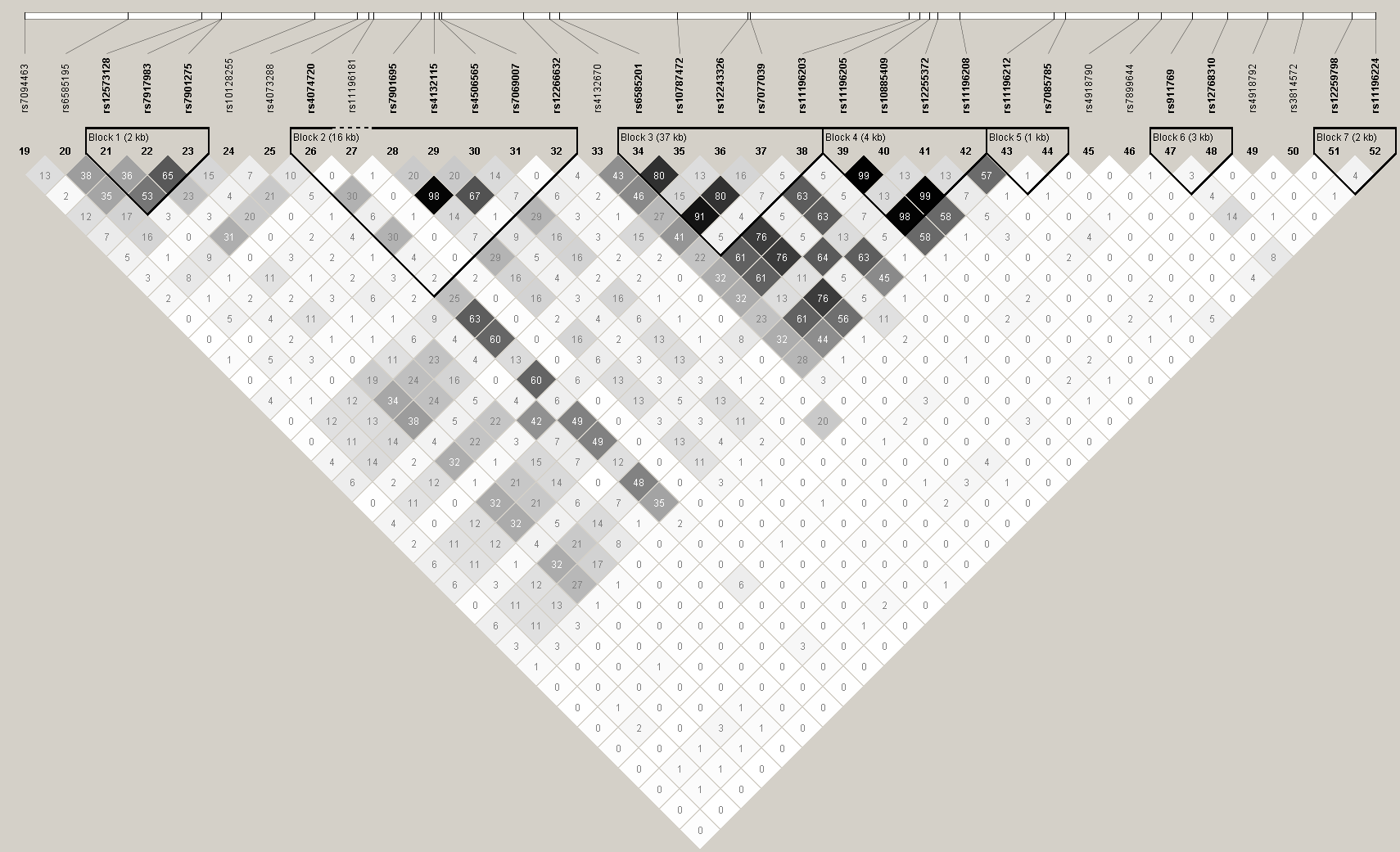
**

^a^ LD estimation performed in HaploView

^b^ Region shown captures the respective ±50kb regions of rs7903146 and rs12255372; markers included are those that passed quality control in ethnic-specific GWAS); rs7903146 not genotyped in Affymetrix 6.0 Platform

**Figure S10. LD structure of *TCF7L2* in MESA European Americans ^a,b^**

**
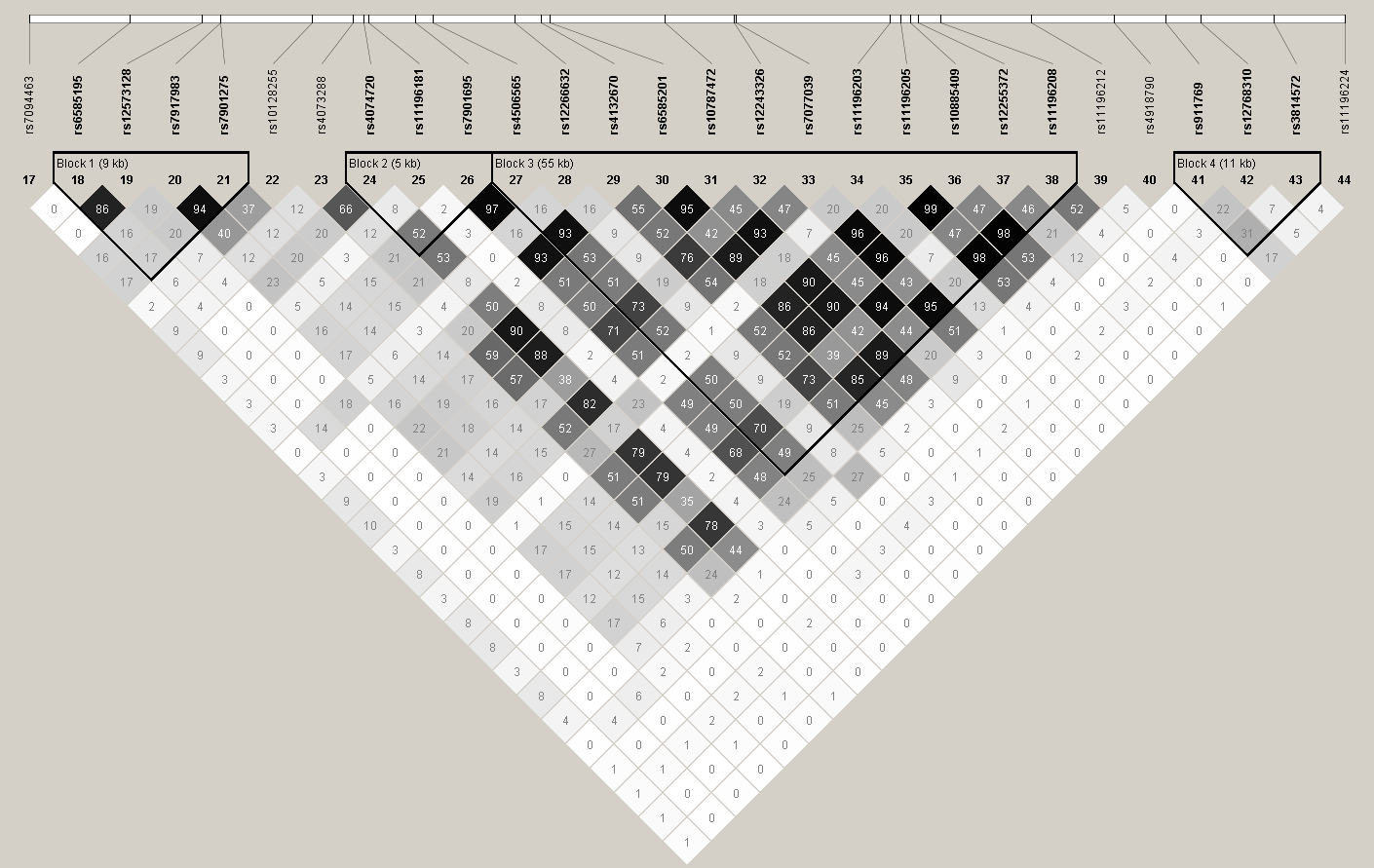
**

^a^ LD estimation performed in HaploView

^b^ Region shown captures the respective ±50kb regions of rs7903146 and rs12255372; markers included are those that passed quality control in ethnic-specific GWAS); rs7903146 not genotyped in Affymetrix 6.0 Platform

**Figure S11. LD structure of *TCF7L2* in MESA Asians ^a,b^**

**
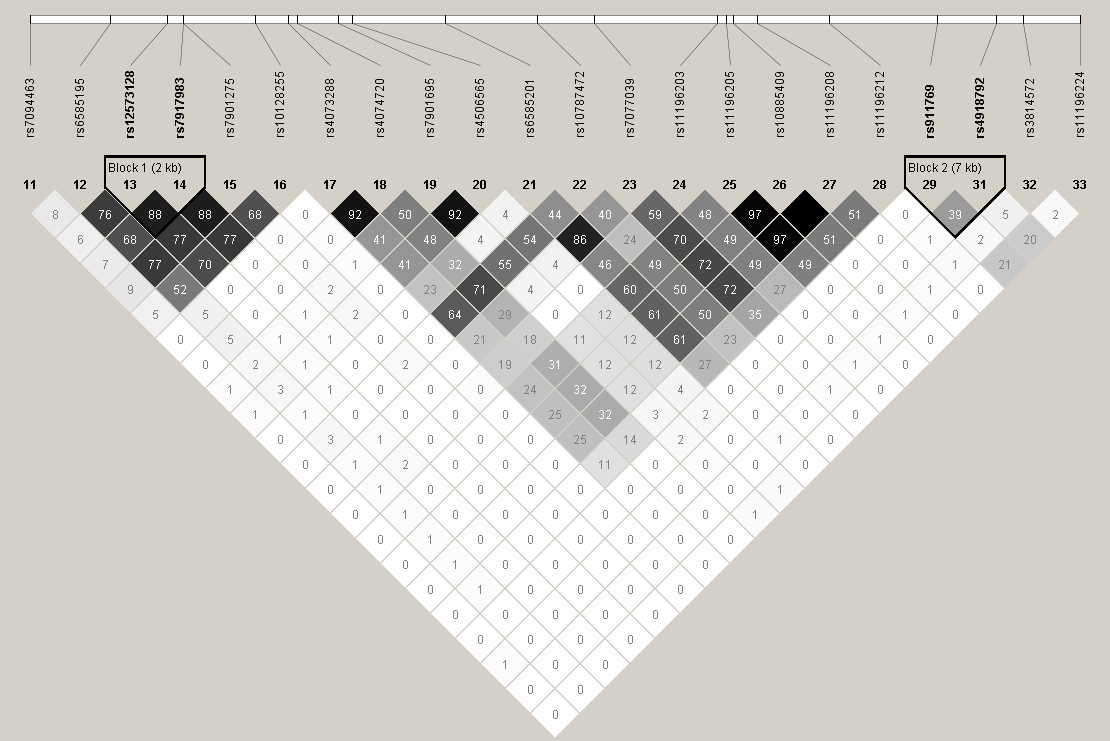
**

^a^ LD estimation performed in HaploView

^b^ Region shown captures the respective ±50kb regions of rs7903146 and rs12255372; markers included are those that passed quality control in ethnic-specific GWAS); rs7903146 not genotyped in Affymetrix 6.0 Platform

**Figure S12. LD structure of *TCF7L2* in WHI Hispanics ^a,b^**


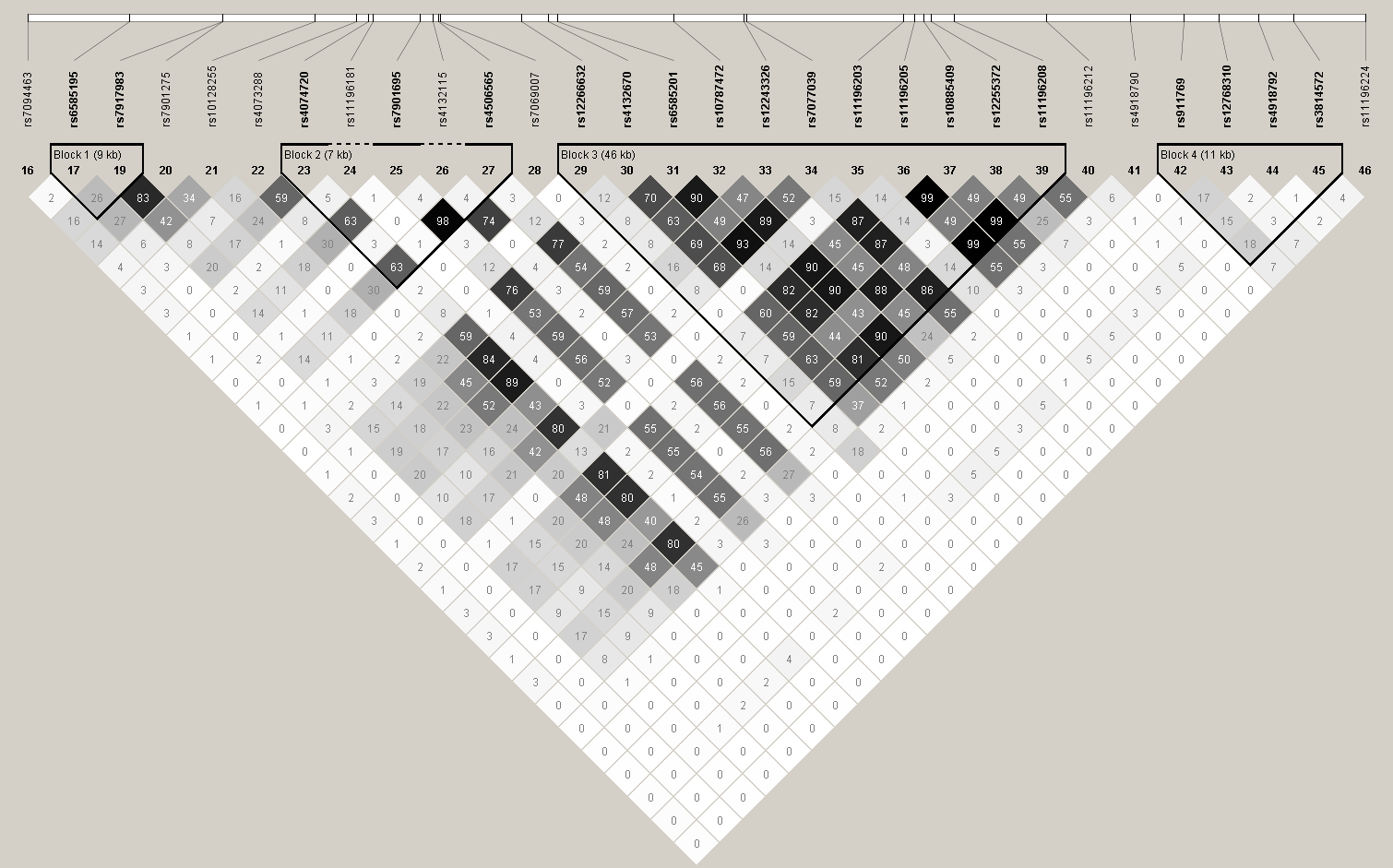


^a^ LD estimation performed in HaploView

^b^ Region shown captures the respective ±50kb regions of rs7903146 and rs12255372; markers included are those that passed quality control in ethnic-specific GWAS); rs7903146 not genotyped in Affymetrix 6.0 Platform

**Figure S13. LD structure of *TCF7L2* in WHI African Americans ^a,b^**

**
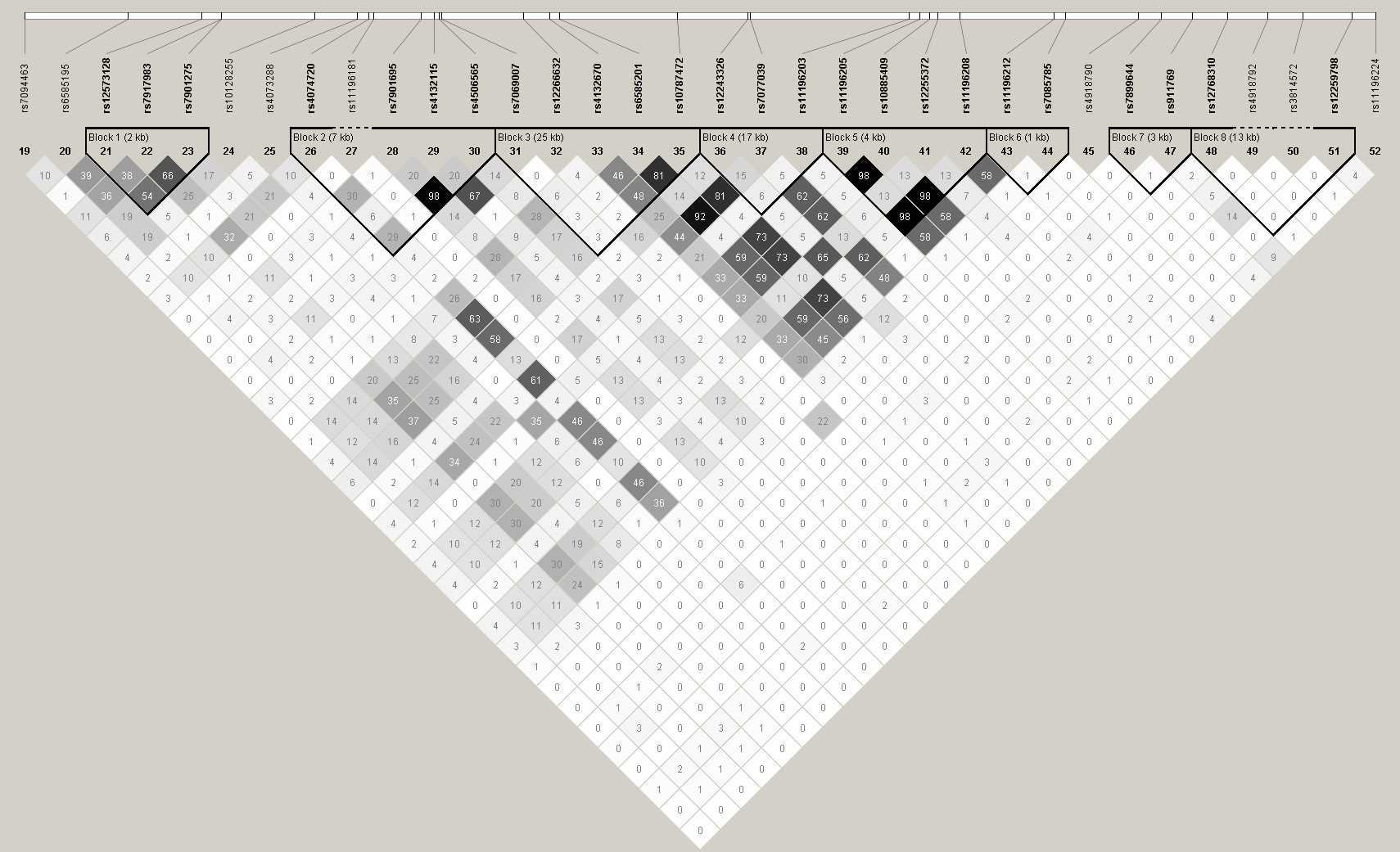
**

^a^ LD estimation performed in HaploView **^a,b^**

^b^ Region shown captures the respective ±50kb regions of rs7903146 and rs12255372; markers included are those that passed quality control in ethnic-specific GWAS); rs7903146 not genotyped in Affymetrix 6.0 Platform
