# Supplementary material for: Multiethnic genome-wide association study identifies ethnic-specific associations with body mass index in Hispanics and African Americans
Source: BMC Genet. 2016 Jun 13;17:78. doi: 10.1186/s12863-016-0387-0 (PMC4907283; doi:10.1186/s12863-016-0387-0)
Supplement: Additional file 17: Figures S14-S15. — PC analyses across the four MESA ethnic groups and across the WHI ethnic groups, plotted in the first two dimensions. (DOCX 622 kb) [file 12863_2016_387_MOESM17_ESM.docx]

# Figure S14. PC analysis across the four MESA ethnic groups, plotted in the first two dimensions

The Multi-ethnic Study of Atherosclerosis (MESA) ascertained subject race and ethnicity via self-report, and grouped subjects accordingly into four racial-ethnic groups: European American, African American, Hispanic or Asian. [For simplicity, in our study, we use the term ‘ethnicity’ to refer to these four racial-ethnic groups]. We performed principal components (PC) analysis across the combined ethnicities using EIGENSTRAT v.3.0 [1]. This figure shows the results of these analyses, plotted in the first two dimensions (PC 1 vs. PC 2) and distinguishing the different ethnic groups. On the whole, these results verify that the MESA ethnic groups cluster together based on genotype data. It is also worth noting that this plot closely matches plots based on the HapMap and Human Genetic Diversity Project populations, as seen in [2]. Though there are subjects that may be considered outliers in each MESA ethnic group, outliers were not removed in the ethnic-specific PC analyses (detailed in the main text) in order to preserve statistical power and to respect the MESA subjects’ self-report that they identify as members of certain ‘racial’ and ‘ethnic’ groups.

##

# Figure S15. PC analysis across the two WHI ethnic groups, plotted in the first two dimensions

The Women’s Health Initiative (WHI) ascertained subject race and ethnicity via self-report, and grouped subjects accordingly into two ethnic groups: Hispanic and African American. [For simplicity, in our study, we use the term ‘ethnicities’ to refer to these two racial-ethnic groups]. We performed principal components (PC) analysis across the combined ethnicities using EIGENSTRAT v.3.0 [1]. This figure shows the results of these analyses, plotted in the first two dimensions (PC 1 vs. PC 2) and distinguishing the different ethnic groups. On the whole, these results verify that the WHI ethnic groups cluster together based on genotype data. Though there are subjects that may be considered outliers in each WHI ethnic group, outliers were not removed in the ethnic-specific PC analyses (detailed in the main text) in order to preserve statistical power and to respect the WHI subjects’ self-report that they identify as members of certain ‘racial’ and ‘ethnic’ groups.

**References.**

1. Price AL, Patterson NJ, Plenge RM, Weinblatt ME, Shadick NA, Reich D: **Principal components analysis corrects for stratification in genome-wide association studies**. *Nat Genet* 2006, **38**(8):904-909.

2. Sikora M, Laayouni H, Calafell F, Comas D, Bertranpetit J: **A genomic analysis identifies a novel component in the genetic structure of sub-Saharan African populations**. *European journal of human genetics : EJHG* 2011, **19**(1):84-88.
