# Supplementary material for: Multiethnic genome-wide association study identifies ethnic-specific associations with body mass index in Hispanics and African Americans
Source: BMC Genet. 2016 Jun 13;17:78. doi: 10.1186/s12863-016-0387-0 (PMC4907283; doi:10.1186/s12863-016-0387-0)
Supplement: Additional file 18: Supplemental Methods. — Details regarding ethnic-specific linear regression model covariates and checks for normality of the response variable. (DOCX 184 kb) [file 12863_2016_387_MOESM18_ESM.docx]

# Supplemental Methods.

**Ethnic-specific linear regression models**

## *Check for normality of the response variable*

Linear regression assumes that error terms are normally distributed, and this is usually guaranteed by a normal distribution of the response variable (body mass index; BMI). Therefore, we examined the distribution of BMI in the discovery and replication datasets through various methods. These included visual inspection of histograms and normal-quantile (QQ) plots **(Additional File 12: Figures S6 and S7)**, calculation of Kurtosis indexes, and formal tests for normality (the Anderson-Darling test).

The BMI distributions were right-skewed (to varying degrees) across all datasets, and the observed BMI values at the upper tails of the distributions deviated from what is strictly expected under the null hypothesis of normality. The skew of the distributions is reflected in the kurtosis indexes. Nevertheless all kurtosis indexes are well within the acceptable range of values (-3 to +3, [1]), suggesting that the untransformed BMI variable may be used as the response variable for linear regression across all datasets. Though this was not supported by the results of the Anderson-Darling tests (all p < 0.05), we would like to note the following: (1) tests for normality become increasingly powerful to detect even the slightest deviations from normality with larger sample sizes; (2) transforming the response variable may compromise the interpretability of the findings; and (3) even if the distribution of the outcome variable is far from normal, the estimators of the linear regression parameters generally have the property of asymptotic normality—their distributions approach normality under very general conditions as the sample size increases [1]. Therefore, we did not transform the response variable.

## *Covariates in MESA*

Variables examined as potential covariates due to their previously-reported associations with BMI were: sex; baseline age (years); education (categorical: highest grade-level completed, grouped into 9 categories ranging from no school to graduate/professional school); income (categorical: total gross income earned by all family members, grouped into 13 categories ranging from <$5,000 to >$100,000); smoking (categorical: never, former, current smokers); arthritis (yes/no); diabetes (yes/no); and moderate and vigorous physical activity (continuous: met-minutes/week). Information regarding these variables was obtained through standardized questionnaires[2].

## *Covariate parameterization in our linear models*

For our analyses, the 9 education categories were collapsed into three: (1) < 12 years of schooling, (2) 12-15 years of schooling (high school graduates and some college), and (3) > 16 years of schooling (college and graduate/professional school graduates). Income was examined as a binary predictor, collapsing the 13 categories into two: high and low income. For this purpose, median household income, as reported by the U.S. Census Bureau, was averaged over the 2000-2002 recruitment period ($41,448). Income categories $40-49,000 and above were defined as high income. Moderate and vigorous physical activity was examined as a categorical predictor since its distribution was strongly right-skewed, with possible outliers, and an F-test suggested that its association with BMI is not linear (p< 0.05). Additionally, a Kruskal-Wallis test revealed that the distribution of moderate and vigorous physical activity differed significantly across the four ethnic groups (p<0.0001), so ethnic-specific cut-points were chosen to divide participants into quartiles. Quartiles for moderate and vigorous physical activity were: 0-1665, 1666-4072.5, 4073-8280, and 8281-45060 met-minutes/week for Hispanics; 0-1305, 1306-2580, 2581-4770, and 4771-30240 met-minutes/week for Asians; 0-2115, 2116-4560, 4561-8625, and 8626-103320 met-minutes/week for African Americans; and 0-2280, 2281-4207.5, 4208-7220, and 7221-56550 met-minutes/week for European Americans.

## *Covariates in WHI*

Covariates were selected based on ethnic-specific regression models built for MESA Hispanics and African Americans: age at baseline (years), education (highest grade completed, grouped into 11 categories ranging from no school to doctoral education), smoking (ever/never), arthritis (yes/no), and diabetes (yes/no). Information regarding these variables was obtained through standardized questionnaires[3].

**References.**

1. Kutner MH: **Applied linear statistical models**, vol. 4. Chicago: Irwin; 1996.

2. Bild DE, Bluemke DA, Burke GL, Detrano R, Roux AVD, Folsom AR, Greenland P, JacobsJr DR, Kronmal R, Liu K: **Multi-ethnic study of atherosclerosis: objectives and design**. *American Journal of Epidemiology* 2002, **156**(9):871-881.

3. WHI-Study-Group: **Design of the Women's Health Initiative Clinical Trial and Observational Study-examples from the Women's Health Initiative**. *Controlled clinical trials* 1998, **19**(1):61-109.
